# Species Divergence vs. Functional Convergence Characterizes Crude Oil Microbial Community Assembly

**DOI:** 10.3389/fmicb.2016.01254

**Published:** 2016-08-12

**Authors:** Yong Nie, Jie-Yu Zhao, Yue-Qin Tang, Peng Guo, Yunfeng Yang, Xiao-Lei Wu, Fangqing Zhao

**Affiliations:** ^1^College of Engineering, Peking University, BeijingChina; ^2^College of Architecture and Environment, Sichuan University, ChengduChina; ^3^State Key Joint Laboratory of Environment Simulation and Pollution Control, School of Environment, Tsinghua University, BeijingChina; ^4^Institute of Engineering (Baotou), College of Engineering, Peking University, BaotouChina; ^5^Beijing Institutes of Life Science, Chinese Academy of Sciences, BeijingChina

**Keywords:** metagenome, microbial community, oil reservoirs, petroleum, methanogenesis, hydrocarbon degradation

## Abstract

Oil reservoirs exhibit extreme environmental conditions such as high salinity and high temperature. Insights into microbial community assemblages in oil reservoirs and their functional potentials are important for understanding biogeochemical cycles in the subterranean biosphere. In this study, we performed shotgun metagenomic sequencing of crude oil samples from two geographically distant oil reservoirs in China, and compared them with all the 948 available environmental metagenomes deposited in IMG database (until October 2013). Although the dominant bacteria and the proportion of hydrogenotrophic and acetoclastic methanogens were different among oil metagenomes, compared with the metagenomes from other environments, all the oil metagenomes contained higher abundances of genes involved in methanogenic hydrocarbon degradation and stress response systems. In addition, a “shape-sorting” manner was proposed for the assembly of microbial communities in oil reservoirs, with the oil reservoir acting as a function sorter to select microbes with special functions from its endemic pool of microorganisms. At the functional level, we found that environmental metagenomes were generally clustered according to their isolation environments but not their geographical locations, suggesting selective processes to be involved in the assembly of microbial communities based on functional gene composition.

## Introduction

Oil reservoirs, which are located deep within the earth, exhibit extreme environmental conditions such as high pressure, high osmosis (due to high salinity or high hydrophobicity), and high temperature (Head et al., 2003). Culture-dependent and culture-independent analyses based on DNA/RNA fingerprints and high throughput 16S rRNA gene sequencing (Li et al., 2006, 2007, 2014; Dahle et al., 2008; Kaster et al., 2009; Pham et al., 2009; Zhang et al., 2012; Gao et al., 2016) have shown that oil reservoirs harbor various microbes. Because of the extreme environmental conditions in oil reservoirs, it is important to understand the functions and assemblies of microial communities in this environment and differences between these microbial communities and those in other environments such as freshwater, seawater, and surface soils. To date, knowledge about functional genes of microbial communities in oil reservoirs is limited despite recent studies on functional genes associated with sulfate reduction, ammonia oxidation, and methanogenesis by analyzing clone libraries (Li et al., 2011; Guan et al., 2013; Liu et al., 2015) and on metagenomes of offshore oil reservoirs of the Norwegian Sea by using shotgun sequencing (Kotlar et al., 2011; Lewin et al., 2014).

Despite the identification of various microbes in oil reservoirs, mechanisms underlying microbial assemblages in these environments remain unclear. Variation in environmental factors such as temperature and pH and biological interactions may influence the microbial communities. The structures of natural microbial communities can be explained by niche-based mechanisms, and differences in the abundance of taxa reflect the differences in the environmental factors. It is supported by numerous evidences that microbial communities change rapidly in response to changes in environmental factors. In contrast, microbial compositions are extremely different in the abundance of rare taxa, sometimes in the high abundant taxa despite similar geochemical conditions in some oil reservoirs (Ren et al., 2011; Kryachko et al., 2012; Tang et al., 2012). These variations in compositions of microbial communities among different oil reservoirs with similar geochemical conditions cannot be explained by environmental factors. It is possible that stochastic processes such as dispersal and colonization play significant roles in structuring microbial communities in these oil reservoirs with similar geochemical conditions. However, this stochastic process cannot explain how oil reservoirs with different geochemical properties show microbial communities with similar taxonomic and functional gene compositions.

Here, we analyzed the crude oil metagenomes from two oil reservoirs in Daqing and Qinghai oil fields in China. These metagenomes were compared with other two crude oil metagenomes from offshore oil reservoirs of Norwegian Sea (Kotlar et al., 2011; Lewin et al., 2014), and 948 environmental metagenomes which were all environmental metagenomes available in IMG database until October 2013, to examine how they differed within and among other environments, and how taxonomic and functional gene compositions were linked to oil reservoirs characteristic conditions.

## Materials and Methods

### Sample Collection

Oil samples were collected from the wellhead of the high temperature Qinghai (QH) oilfield located in the Tibetan Plateau situated in the northwest of China and from the wellhead of the mesophilic Daqing oilfield (DQ) located in the northeast of China (Supplementary Figure S1). The distance between the two sampling sites is more than 2,500 km. Blocks of Qinghai and Daqing oilfield from where the oil samples were obtained have been subjected to water flooding for 15 and >30 years, respectively. Samples were collected directly from sampling valves located on the wellheads before the oil entered production facilities such as treatment station for separating water, oil, gas, and sand (Supplementary Figure S2). To avoid contamination, sampling cans were washed, sealed, and sterilized by heating at 121°C for 20 min. The sampling valves were also washed and sterilized with 70% alcohol immediately before sampling. During sampling, an initial 20-L sample was discarded before collecting the oil samples in the sterilized cans. Once the cans were completely filled, they were sealed using screw caps, stored at <5°C, and sent to our laboratory within 2 days. Further analyses were performed immediately. Since the wellheads were directly connected to the working layers in reservoirs, the microbial community in the crude oil samples should reflect the microbial compositions in the reservoirs.

### DNA Extraction, Sequencing, and Analysis

DNA was extracted from the samples as described previously (Kryachko et al., 2012) and the genomic libraries for Illumina sequencing were generated according to the Illumina paired-end library preparation protocol. The average size of the library fragments was 200 bp for both QH and DQ, while the average of the insert size was 190 bp for QH and DQ. The libraries were sequenced using HiSeq 2000 (Illumina, San Diego, CA, USA) with 2 × 100 bp paired-end sequencing.

For taxonomic analysis, low quality paired-end reads were removed using Illumina CASAVA pipeline with default parameters, and the remaining reads were compared against the NR database by using BLASTX to estimate functional and taxonomic composition and abundance as described before (Wang J. et al., 2013). MEGAN software (Huson et al., 2011) was used to parse the results of BLASTX and to estimate microbial composition using the lowest common ancestor (LCA) algorithm. Briefly, each read matching the sequence of any gene was placed onto the LCA node of those species in the NCBI taxonomy that are known to have that gene, and the taxonomic information of the LCA node was retrieved. To confirm the results, the 16S rRNA genes were also extracted from the reads based on hidden Markov Models (HMMs; Huang et al., 2009), and classified using the RDP classifier (Cole et al., 2009).

For metagenome assembly and gene prediction, after quality filtering of the raw reads, the remaining paired-end reads were used to build a *de novo* assembly by using SOAPdenovo (Luo et al., 2012) with default settings. Open reading frames (ORFs) of assembled contigs were predicted using MetaGeneMark program (Zhu et al., 2010). The predicted ORFs were compared against the NCBI NR database (downloaded from NCBI in September, 2012) of non-redundant protein sequences, the Kyoto Encyclopedia of Genes and Genomes (KEGG) orthologous (KOs) database (Kanehisa et al., 2008), and Clusters of Orthologous Groups (COG) database (Tatusov et al., 2003) to assess the functional classification. For searching genes encoding alkane 1-monooxygenase (AlkB), cytochrome P450 CYP153 family of alkane hydroxylases (CYP153), long-chain alkane monooxygenase (LadA), flavin-binding monooxygenase (AlmA) involved in aerobic *n*-alkane metabolism, and hydrocarbon succinate synthases (AssA/BssA) involved in anaerobic hydrocarbon degradation in the two metagenomes, reference sequences were downloaded from GenBank (Supplementary Table S1). Genes encoding AlkB and CYP153 were annotated as described previously (Nie et al., 2014). Two crude oil metagenomes NorOil1 (Kotlar et al., 2011) and NorOil2 (Lewin et al., 2014) from offshore oil reservoirs of Norwegian Sea were also analyzed using the same methods.

To identify special genes and pathways in the metagenomes of the oil reservoirs, all the published 1,233 metagenomic datasets from the IMG database (until October, 2013) were downloaded and compared. After removing metagenomes that were not environmental samples, or were without detailed information about the sampling sites, 948 metagenome datasets were selected for analysis. Both the oil metagenomes and all the reference metagenomes were clustered using Cluster 3.0 program (de Hoon et al., 2004) with average linkage method based on the relative abundance of KEGG genes after normalization according to *z*-score. To identify specific functions of adaptive importance in different samples, genes in different regions were affiliated to KEGG pathways. Next, abundance of genes in different pathways was normalized using the *z*-score and was clustered. Bray–Curtis dissimilarity was calculated using Vegan: community ecology package (version 1.8–6) in R statistical program 2.9.1 (R Development Core Team). Based on the KO abundance, principal component analysis (PCA) was performed to evaluate similarity among various metagenomes by using the Vegan package in the R statistical program.

The metagenomics data have been submitted to NCBI SRA and are accessible under the BioProject identifier PRJNA251580.

## Results

### Microbial Community Structure in Oil Samples

**FIGURE 1:**
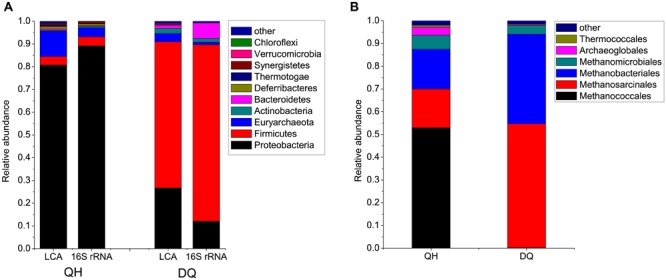
**Microbial communities in crude oil samples. (A)** Microbial compositions at the phylum level. The phyla with average abundances in Qinghai (QH) and Daqing oilfield (D) Q more than 0.2% were shown, and the remaining phyla were combined as “other” in the figure. LCA, the reads were compared against the NR database by using BLASTX. The MEGAN software was used to parse the results of BLASTX and to estimate microbial composition and abundance. 16S rRNA, microbial compositions at the phylum level based 16S rRNA genes extracted from the reads and classified using RDP classifier. **(B)** Archaeal compositions at the order level.

After filtering low-quality reads, 68,937,779 and 68,362,793 read pairs were generated from the metagenomes of Qinghai (QH) and Daqing (DQ) oilfields, respectively. Approximately 72.2% reads obtained from the QH metagenome were assigned to bacteria and archaea (Supplementary Figures S3 and S4); of these, approximately 93% reads were assigned to 34 known phyla (**Figure 1**; Supplementary Figure S4; Supplementary Table S2). Approximately 73.6% reads obtained from the DQ metagenome were assigned to bacteria and archaea (Supplementary Figure S4); of these, approximately 93% reads were assigned to 35 known phyla (**Figure 1**; Supplementary Figure S4; Supplementary Table S2). However, approximately 36.2% and 45.4% reads obtained from the QH and DQ metagenomes, respectively, that were assigned to known phyla could not be assigned to known genera (Supplementary Figure S4). The rarefaction analysis of reads based on the taxonomic information showed that a reasonable number of individual genomes were sampled, with the coverage over 99% both in QH and DQ at the genus level (Supplementary Figure S5).

The QH metagenome predominantly included bacteria belonging to *Proteobacteria*, which accounted for 80.8% of the total reads assigned to known phyla (**Figure 1**). Of the *Proteobacteria*, *Gammaproteobacteria* was the most dominant bacterial class, with genera such as *Pseudomonas* (54.4%), *Acinetobacter* (1%), and *Marinobacter* (1.3%) (Supplementary Figure S6). The remaining microbes mainly belonged to *Euryarchaeota* (11.1%), *Firmicutes* (3.8%), and *Deferribacteres* (1.2%). The QH metagenome included methanogenic archaea (47 genera) such as hydrogenotrophic *Methanococcus*, *Methanothermococcus*, *Methanobacterium*, *Methanoculleus*, and *Methanosarcina* that could produce methane from H_2_, acetate or methyl compounds (Supplementary Figure S7; Supplementary Table S3). The DQ metagenome predominantly included bacteria belonging to *Firmicutes* (64.2%) and *Proteobacteria* (26.7%), namely, *Anoxybacillus* (37.2%), *Geobacillus* (2.8%), *Bacillus* (1.1%), *Pseudomonas* (6.4%), and *Brevundimonas* (0.9%) (Supplementary Figures S6 and S8). Microbes belonging to *Euryarchaeota* (3.6%), *Actinobacteria* (2.2%), and *Bacteroidetes* (1.2%) were also detected in QH metagenome. The DQ metagenome included methanogenic archaea such as acetoclastic *Methanosaeta* (Supplementary Figure S7; Supplementary Table S3). *Poribacteria* and *Fibrobacteres* were only detected in the DQ metagenome, and *Nanoarchaeota* were only detected in the QH metagenome; however, abundances of these bacteria were very low in both the metagenomes (Supplementary Table S2).

To confirm the results, the 16S rRNA genes were also identified from metagenome reads and classified using the RDP classifier. A total of 3,210 and 1,792 16S rRNA gene fragments were extracted from the reads from QH and DQ metagenomes, respectively. The results revealed that the taxonomic distributions were similar to those from BLASTX-based classification (**Figure 1**).

### Genes Related to Petroleum Degradation Pathways in Oil Reservoirs

**FIGURE 2:**
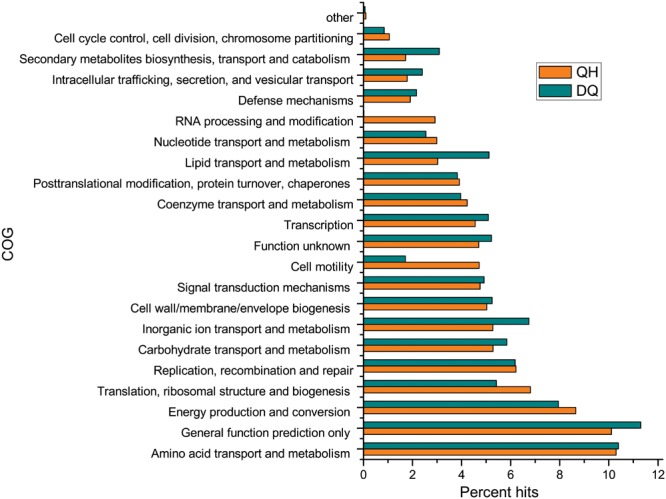
**Functional composition of the metagenomes from crude oil samples according to the clusters of orthologous groups (COG) database.** Open reading frames (ORFs) of the assembled contigs were predicted using MetaGeneMark program and were compared against the COG database to assess the functional classification.

In all, 184,871 and 305,582 ORFs were found in the QH and DQ metagenomes, respectively. Of these, 54.9 and 60.0% ORFs, respectively, were assigned to COG database (**Figure 2**) and 34.3 and 34.8% ORFs, respectively, were assigned to KOs database (Supplementary Figure S9). Functional community profiles of the QH, DQ, NorOil1, and NorOil2 metagenomes were created based on KEGG annotations. KEGG annotations determined genes involved in complete hydrocarbon metabolism in both QH and DQ metagenomes; however, these genes showed remarkably different taxonomic affiliations. Both QH and DQ metagenomes contained high abundance of genes in lipid metabolism. For example, 129 and 395 genes for 3-oxoacyl-(acyl-carrier-protein) reductase (KO: K00059) were detected in the QH and DQ metagenomes, respectively. These genes were similar to sequences from *Pseudomonas* and *Marinobacter* in the QH metagenome and *Anoxybacillus* and *Pseudomonas* in the DQ metagenome. These data were consistent with the microbial taxonomic compositions of the two metagenomes. Both QH and DQ metagenomes contained glycosyltransferase genes essential for glycolipid and lipopeptide biosynthesis (Supplementary Tables S4 and S5). Moreover, both QH and DQ metagenomes contained high abundance of genes in lipid metabolism. For example, 129 and 395 genes for 3-oxoacyl-(acyl-carrier-protein) reductase (KO: K00059) were detected in the QH and DQ metagenomes, respectively. These genes were similar to sequences from *Pseudomonas* and *Marinobacter* in the QH metagenome and *Anoxybacillus* and *Pseudomonas* in the DQ metagenome. These data were consistent with the microbial taxonomic compositions of the two metagenomes.

Alkane hydroxylases act in the first step of aerobic hydrocarbon degradation. Genes encoding alkane hydroxylase (EC: 1.14.15.3), alcohol dehydrogenase (EC: 1.1.1.1), and aldehyde dehydrogenase (EC: 1.2.1.3) were detected in both the metagenomes, indicating complete alkane degradation through β-oxidation pathway for fatty acid degradation. The QH and DQ metagenomes included ten and 46 genes encoding alkane 1-monooxygenase (AlkB; EC: 1.14.15.3), three and 66 genes encoding cytochrome P450 CYP153 family of alkane hydroxylases (EC: 1.14.15.3), 10 and 67 genes encoding long-chain alkane monooxygenase (LadA; Feng et al., 2007), and 36 and 149 genes encoding protein *n*-alkane metabolism A (AlmA; Throne-Holst et al., 2007), respectively (Supplementary Figures S10–S13). These results indicated that the DQ metagenome included more diverse genes encoding alkane hydroxylases than the QH metagenome. Genes encoding aerobic alkane hydroxylases were not detected in the NorOil metagenomes. Besides genes involved in aerobic alkane degradation, 16, 14, 18, and 18 genes encoding hydrocarbon succinate synthases (AssA/BssA) were present in the QH, DQ, NorOil1, and NorOil2 metagenomes, respectively. Proteins encoded by these genes activate anaerobic hydrocarbon degradation followed by benzoyl-CoA or fatty acid metabolism (Widdel and Rabus, 2001). Further, *assA*/*bssA* genes were detected with similarities to sequences from *Desulfotomaculum*, *Anaerofustis*, *Desulfatibacillum*, *Pelobacter*, *Bacteroides*, *Pseudomonas*, *Anaerobaculum*, *Spirochaeta*, and *Thermococcus* in the QH metagenome; *Desulfatibacillum*, *Desulfoglaeba*, *Desulfosporosinus*, *Desulfotomaculum*, *Anoxybacillus*, *Pseudomonas*, and *Sanguibacter* in the DQ metagenome; *Desulfovibrio*, *Pelobacter*, *Clostridium*, and *Thermovirga* in the NorOil1 metagenome; and *Desulfoglaeba*, *Desulfobacula*, *Geobacter*, *Azoarcus*, *Aromatoleum*, *Thauera*, and *Clostridia* in the NorOil2 metagenome (Supplementary Figure S14).

Bifunctional carbon monoxide dehydrogenase/acetyl-CoA synthase (CODH/ACS, EC: 2.3.1-, 1.2.99.2, 1.2.7.4) is known as one of the key enzymes in Wood-Ljungdahl (W-L) pathway (Ragsdale, 2007). Here, we detected the genes encoding CODH/ACS in the QH and DQ metagenomes, to estimate the distribution of bacteria with W-L pathway and syntrophic acetate oxidation (SAO) potentials. In all, 79 and 60 reads matching the genes encoding CODH/ACS were detected in the QH and DQ metagenomes, respectively. Only 44 reads in the QH metagenome were assigned to classes *Clostridia*, *Deltaproteobacteria*, and *Synergistia* and 20 reads in the DQ metagenome were assigned to classes *Alphaproteobacteria*, *Deltaproteobacteria*, and unclassified *Poribacteria* (Supplementary Figure S15).

### Clustering of Metagenomes of Oil Reservoirs

Taxonomic and functional compositions of QH, DQ, NorOil1, and NorOil2 metagenomes of oil reservoirs were compared. Bray–Curtis dissimilarity analysis indicated that the metagenomes of the four oil reservoirs (QH, DQ, NorOil1, and NorOil2) showed 58.4% average dissimilarity in taxonomic compositions and 32.2% average dissimilarity in functional compositions (KEGG). These results showed that despite large differences in taxonomic compositions across metagenomes of oil reservoirs, they shared many genes, especially enriched or reduced genes with the same or similar functions, from different species that constituted the core functions of these environments.

**FIGURE 3:**
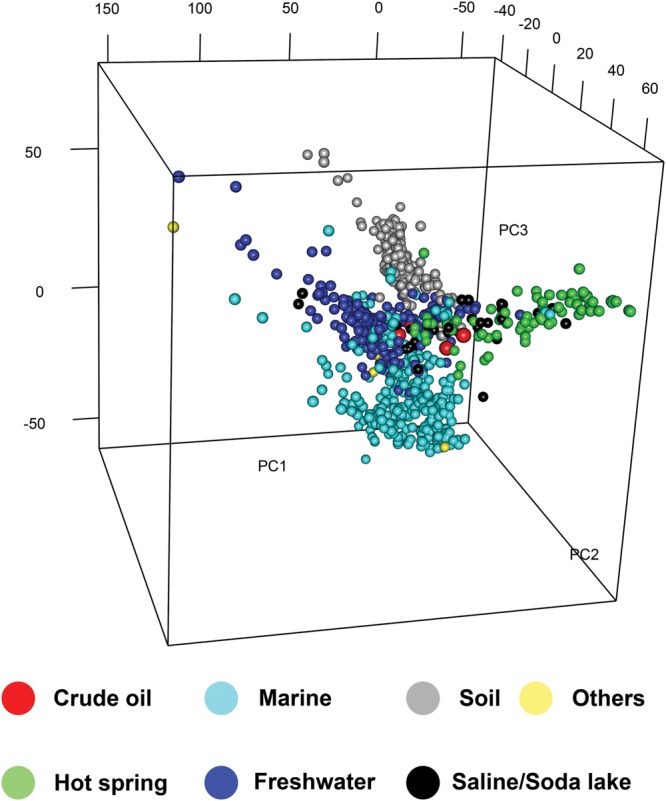
**Principal component analysis (PCA) of all the metagenomes based on the KO abundance.** Four metagenomes from oil samples and the 948 environmental metagenomes deposited in the IMG database were used for PCA analysis using the KO abundances (For representative environmental samples, see Supplementary Tables S6–S9). Different colors denote different isolation environments.

Hierarchical clustering of KO abundances was performed for metagenomes from the four oil samples and the 948 environmental metagenomes deposited in the IMG database (until October 2013). PCA analysis was performed using the KO abundances to analyze the relationship among the functional compositions of metagenomes from different environments, which also classified all the metagenomes into four groups according to their isolation environments (**Figure 3**). Notably, all the environmental metagenomes did not cluster according to their geographical locations but clustered according to their isolation environments (Supplementary Figure S16A), which formed four major clusters (cluster I to cluster IV). Generally, metagenomes from soil, freshwater, and marine samples were clustered, respectively, and were separated from the metagenomes from oil reservoirs and other extreme environments (**Figure 3**; Supplementary Figure S16A). In cluster I, the metagenomes from the QH, DQ, NorOil1, and NorOil2 reservoirs were clustered with metagenomes from other extreme environments (Supplementary Table S6). Cluster II included metagenomes mainly from terrestrial soil environments (Supplementary Table S7). Cluster III included metagenomes mainly from marine environments (Supplementary Table S8) and cluster IV included metagenomes from freshwater environments (Supplementary Table S9). These different metagenome clusters showed different enriched functional genes and were viewed as four “hot” gene pools, denoted as groups A–D (Supplementary Figure S16B). In these gene groups, Groups A, B, C, and D corresponded to Clusters I, II, III, and IV, respectively, in the heat map (Supplementary Figure S16A). Of these enriched genes, genes in group A were associated with 43 pathways, including pathways of carbon metabolism, methane metabolism, amino acid biosynthesis (Supplementary Figure S16B; Supplementary Tables S10 and S11). For example, 146 KOs in group A (5.1% of the total KOs in group A) were assigned to the carbon metabolism pathway, including genes encoding CODH/ACS involved in W-L pathway. In contrast, other genes in carbon fixation, such as RuBisCO in Calvin cycle and ATP-citrate lyase in the reductive tricarboxylic acid cycle (Atomi, 2002), were less abundant in group A and in the metagenomes of oil reservoirs. We also identified pathways in which genes in groups A and B were both highly distributed. These genes were mainly related to nitrogen metabolism, two-component systems (TCSs), ABC transporters, and bacterial chemotaxis (Supplementary Figure S16B; Supplementary Table S11).

### Specific Functional Profiles of Metagenomes of Crude Oil Reservoirs

We closely examined the enriched gene pool in group A. Methanogenesis-related functions were among the enriched pathways. In all, 93 KOs of methane metabolism were detected, constituting both complete hydrogenotrophic (KEGG module M00567) and acetoclastic (M00357) methanogenic pathways. The QH and DQ metagenomes included 78 and 27 reads, respectively, matching methyl-coenzyme M reductase alpha subunit (McrA), a key enzyme for methanogenesis and specific marker of methanogens (Luton et al., 2002; Supplementary Figure S17). *mcrA* genes similar to those from *Methanococcales* were dominant in the QH metagenome, accounting for 16.7% of the total *mcrA* genes. *Methanobacteriales* and *Methanosarcinales* similar *mcrA* genes were dominant in the DQ metagenome, accounting for 37.0 and 33.3% of the total *mcrA* genes, respectively. These results also showed that the DQ metagenome had more genes with similarities to sequences from *Methanosarcinales* and *Methanobacteriales*. Further, nitrogen fixation-related genes *nifDKH* (K02586, K02591, and K02588) were similar to the *nif* genes from methanogens such as *Methanococcales* (144/289 reads), *Methanobacteriales* (22/289 reads), and *Methanosarcinales* (19/289 reads) in the QH metagenome and *Methanobacteriales* (13/90 reads), *Methanomicrobia* (5/90 reads) in the DQ metagenome (Supplementary Figure S18).

In all, 15 complete TCSs such as PhoR-PhoB, KinB-AlgB, YesM-YesN, and VicK-VicR (Supplementary Table S12); 19 complete ABC transporter systems (Supplementary Table S13) were detected in the enriched KOs obtained from the QH and DQ metagenomes. TCSs are important for microbes to adapt to the extreme environment in oil reservoirs, which is anaerobic and hydrophobic, lacks phosphates and amino acids, and contains hydrocarbons as carbon sources and solvents. Genes of TCSs were similar to sequences from *Methanococcaceae*, *Methanosarcinaceae*, *Clostridiales*, *Proteobacteria*, and *Deferribacteres* in the QH metagenome and *Anoxybacillus* in the DQ metagenome. The complete ABC transporter systems in the metagenomes of the oil reservoirs included membrane transport for glutamine and cysteine. However, transporters for L-arabinose, lactose, and oligogalacturonide and for multiple sugars were absent.

Although not enriched, many genes related to bacterial osmotic stress responses, including those encoding transcriptional regulators and proteins involved in compatible solute transport and synthesis, were present in the metagenomes of the oil reservoirs (Supplementary Table S14), which was similar to that observed in other extreme environments.

## Discussion

**FIGURE 4:**
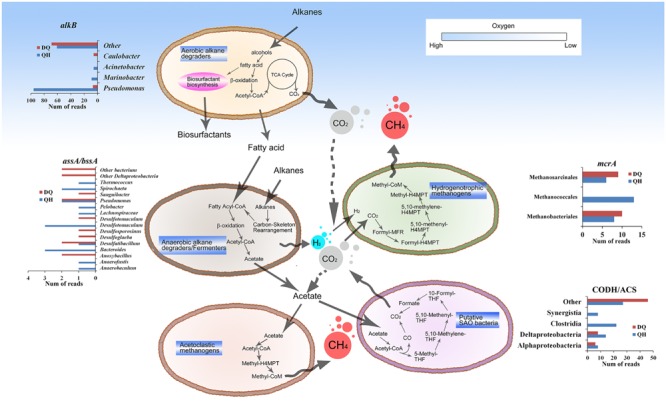
**General microbial metabolic pathways in the oil reservoirs.** The pathways in the oil reservoirs were reconstructed by functional genes annotation. Briefly, hydrocarbons were oxidized aerobically in the shallow subsurface oil reservoirs or anaerobically in the deep subsurface oil reservoirs. The metabolites such as acetate and H_2_ and were linked to methanogenesis from CO_2_ reduction, acetoclastic methanogenesis, or syntrophic acetate oxidation (SAO). The genes involved in the pathways were similar with sequences from different taxa, the abundances of which were listed aside the pathway.

Although oil reservoirs exhibit extreme conditions to support life, accumulating evidence has indicated that oil reservoirs are complex ecosystems comprising numerous microbes (Li et al., 2007; Dahle et al., 2008; Kaster et al., 2009). To adapt to these ecosystems, microbes have developed compatible functions such as enriched systems for carbon metabolism, methane metabolism, stress response, sensing, and transport. Enrichment of genes involved in carbon metabolism pathways might be because of the special requirements of carbon metabolism in these harsh environments. For example, although petroleum constituents are hydrophobic and toxic, they are the only potential organic carbon source for microbes in oil reservoirs and contribute to the enrichment of genes involved in both aerobic and anaerobic alkane degradation pathways, methane metabolism, TCSs, and ABC transporter systems. Both QH and DQ metagenomes contained genes essential for glycolipid and lipopeptide biosynthesis (Supplementary Tables S4 and S5), which could help bacteria to access hydrophobic oil constitutes (Wang X.B. et al., 2013). Enriched genes encoding AlkB, CYP153, LadA, and AlmA for aerobic alkane degradation were detected in the QH and DQ metagenomes but were absent in the NorOil1 or NorOil2 metagenomes. This might be because the DQ and QH reservoirs have been subjected to water flooding for more than 10 years. This may have provided small amount of oxygen through the injected water into the oil reservoirs and that may have stimulated aerobic alkane metabolism. Oxygen can also be provided by oxygen-bearing meteoric water, which results in the aerobic degradation of crude oil in shallow subsurface oil reservoirs (Aitken et al., 2004). However, anaerobiosis is dominant in oil reservoirs (Head et al., 2003; Aitken et al., 2004; Dolfing et al., 2007; Jones et al., 2008). The metabolites such as organic acids generated during aerobic hydrocarbon degradation at the shallow subsurface could be further utilized in the deep surface of the oil reservoirs. Besides, anaerobic degradation of hydrocarbons in deep subsurface oil reservoirs are linked to methanogenesis via acetate or H_2_/CO_2_ (**Figure 4**; Dolfing et al., 2007). The acetate and H_2_/CO_2_ could be linked to several reactions and they might form a complex regulating network (**Figure 4**) which is influenced by factors such as H_2_ and CO_2_ concentration and temperature. SAO bacteria were thought using the reversal of W-L pathway to convert acetate to CO_2_/H_2_ that could be subsequently utilized by hydrogenotrophic methanogens (Hattori, 2008). Moreover, the enzyme activities in W-L pathway such as CO dehydrogenase, were detected in SAO bacteria (Hattori et al., 2005). The high abundance of hydrogenotrophic methanogens such as *Methanococcales*, *Methanobacteriales*, and *Methanomicrobiales* in the metagenomes of the oil reservoirs (**Figure 1**; Supplementary Figure S17), and the low concentration of detectable H_2_ in oil reservoirs and microcosms of methanogenic hydrocarbon degradation (Mayumi et al., 2011) suggest a quick consumption of H_2_ by hydrogenotrophic methanogens and low possibility of homoacetogenesis in oil reservoirs. The low concentration of H_2_ could also benefit SAO bacteria (Dolfing et al., 2007; Kotlar et al., 2011), which convert acetate to H_2_ and CO_2_. Moreover, at high temperatures, acetoclastic methanogenesis becomes thermodynamically less favorable (Dolfing et al., 2007). Thus, at high temperatures, hydrogenotrophic methanogens is dominant. Therefore, hydrogenotrophic methanogens such as *Methanothermococcus* were dominant in the NorOil1 (at 85°C) and NorOil2 (at 83°C) metagenomes (Kotlar et al., 2011; Lewin et al., 2014), and the proportion of *Methanococcus* was higher in the QH metagenome while that of *Methanosaeta* was higher in the DQ metagenome. These results were consistent with those reported by Pham et al (Pham et al., 2009) who showed that acetoclastic *Methanosaeta* were dominant in some mesophilic oil reservoirs.

In contrast to the abundance of hydrocarbons, absence of saccharides in oil reservoirs should contribute to the low abundance of genes associated with sugar metabolism in the metagenomes of oil reservoirs. Although genes such as RuBisCO in Calvin cycle, and ATP-citrate lyase in the reductive tricarboxylic acid cycle were detected in low abundances, the detection of genes in WL pathway suggested the existing of chemoautotrophic microbes in oil reservoirs. Besides metabolism of hydrocarbons, this pathway could also provide organic carbon sources for other microbes. Furthermore, due to the absence of amino acids in oil reservoirs, microbes in these environments evolved effective systems to synthesize and transport amino acids for protein translation, as observed in group A (cluster I, Supplementary Figure S16A). Interestingly, *nif* was mainly enriched in methanogens in group A. It was not unusual because such genes have also been detected in some methane seep and consuming environments (Dekas et al., 2009; Miyazaki et al., 2009). Nitrogen-fixing archaea in these environments might use nitrogen fixation to overcome the scarcity of bioavailable nitrogen and may serve as the source of nitrogen for microbial communities (Dekas et al., 2009). Due to the low concentration of ammonia in oil reservoirs, it was suggested the important role of methanogens in the nitrogen cycle in oil reservoirs.

Besides hydrocarbon metabolism-related genes, microbes in oil reservoirs have developed special systems to adapt to crude oil habitats, to survive, to obtain nutrients and metabolize it, and to escape toxic compounds and high osmosis. For example, enriched VicR/VicK TCS, which mediates exopolysaccharide biosynthesis and biofilm formation (Fabret and Hoch, 1998; Winkler and Hoch, 2008), and LiaS/LiaR TCS, which provides resistance against organic solvents (Mascher et al., 2004), are beneficial for microbes to mitigate the impact of osmosis from petroleum hydrophobicity and to promote the survival of microbial communities. AtoS/AtoC can induce the bacterial growth in short-chain fatty acids, and positively regulate the levels of poly-(R)-3-hydroxybutyrate (PHB; Kyriakidis and Tiligada, 2009). PHB is a member of the polyhydroxyalkanoate (PHA) which serves as the intracellular carbon and energy storage compounds and enhances the survival of bacteria, usually when an essential nutrient such as nitrogen or phosphorus is limited in the presence of excess carbon source (Anderson and Dawes, 1990). These functions are important for microbes to survive in extreme environments of oil reservoirs, where the organic carbon sources, i.e., hydrocarbons are abundant, but the bioavailable nitrogen and phosphorus are limited.

High functional similarity observed among the metagenomes of the oil reservoirs was also observed among those of other environments. Functional profiles of all the 948 environmental metagenomes deposited in the IMG database (until October 2013) showed that metagenomes from soil, freshwater, marine, and extreme environments had their own special functions, irrespective of their geographical location (**Figure 3**; Supplementary Figure S16). This was consistent with the results of recent studies, which showed that microbes in similar environments selected similar functions to adapt to their surroundings (Nemergut et al., 2013). For example, higher availability of aromatic compounds such as aromatic carboxylic humic acids in soil (Van Trump et al., 2011) plays an important role in enriching genes involved in aerobic degradation of aromatic compounds (group D; Supplementary Figure S16B). In marine environments, high concentrations of nitrate and nitrite in euphotic and mesopelagic zones below the nitracline (Shi et al., 2010) and extreme oligotrophy in deep sea (Smedile et al., 2014) are important for enriching genes involved in nitrogen metabolism, bacterial chemotaxis, and flagellar assembly (group B; Supplementary Table S11). Therefore, it is not surprising that many marine bacteria are motile, with the proportion of motile cells being as high as 20–60% (Stocker, 2012). Consequently, specific environmental factors to the four environments resulted in the selection of characteristic microbial functions and clustering of metagenomes from these environmental (**Figure 3**). Similarly, genes related to methane metabolism were enriched in the metagenomes of the oil reservoirs. This was consistent with the finding that methanogenic anaerobic degradation of crude oil is an important process in subsurface petroleum reservoirs, which distinguishes oil reservoirs from other environments (Jones et al., 2008).

**FIGURE 5:**
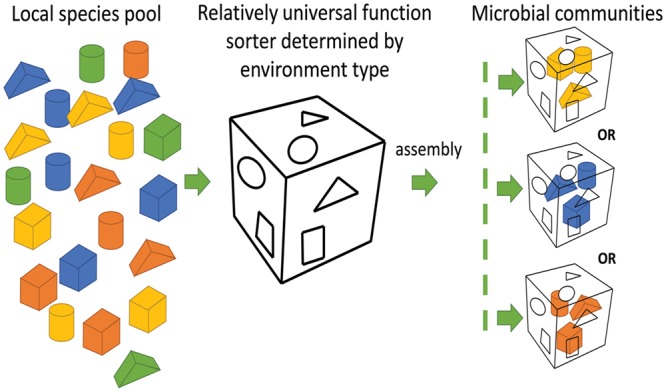
**Microbial community assembly in a shape sorter manner.** Right-shaped blocks (genes with similar functions), despite their color (different taxa), have equal possibility of passing the shape filter (the characteristic of an environment) and getting into the sorter, microbes with genes that match the environmental functional filters, irrespective of their taxa, can be selected stochastically.

Microbial compositions at the taxonomic level varied remarkably among oilfields. Microbial compositions of the metagenomes from offshore oil reservoirs of Norwegian Sea (NorOil1 and NorOil2; Kotlar et al., 2011; Lewin et al., 2014) which were located in the same geographical region but were physically separated, were remarkably different from the metagenomes of the QH and DQ reservoirs. *Delta/Epsilon-proteobacteria* and methanogens were abundant in the NorOil1 metagenome (Kotlar et al., 2011). However, archaea were the most dominant microbes in the NorOil2 metagenome (Lewin et al., 2014). In contrast, *Pseudomonas* and *Anoxybacillus* were dominant in the QH and DQ, respectively (**Figure 1**). Microbial compositions at the taxonomic level were remarkably different even between these oil reservoirs. However, functional compositions, i.e., metabolic pathways and functional genes, were common among geographically distant oil reservoirs and extreme environments such as hot spring and soda lakes. Bray–Curtis dissimilarity analysis also confirmed the results that despite large differences in taxonomic compositions across metagenomes of oil reservoirs, they shared many genes, especially enriched or reduced genes with the same or similar functions, from different species. These phenomena may be attributed to the mechanisms underlying the assembly of microbial communities. The popular niche theory suggests that the structure of a microbial community is shaped mainly by factors such as environmental conditions, competition, and niche differentiation. Environment and microbial interactions dominate this process (Ofiteru et al., 2010). In contrast, neutral theory proposes that species in environments are ecologically equivalent and that the structures of microbial communities are determined randomly (Woodcock et al., 2007). However, these two theories can only explain a part of our results. The niche theory explained the selection of similar microbial functions by similar environments (**Figure 3**) but could not explain the remarkable difference in taxonomic microbial compositions. In contrast, the neutral theory explained the variation in the distribution of microbes at the taxonomic level among different oil reservoirs, which was consistent with the stochastic model of community assembly. However, this theory could not explain the clustering of samples from four typical environments. Divergence of species and convergence of functional compositions observed in the metagenomes of the oil reservoirs suggested that the assembly and structure of bacterial communities may not be based on species but may be based on functional genes determined by the respective environments, which was consistent with the findings of a previous study (Burke et al., 2011). In this aspect, the assembly of microbial communities in oil reservoirs was affected by the effects of the deterministic assembly processes in a “shape-sorting” manner based on the functional compositions. Like a shape sorter that contains different shape filters and that only allows blocks with the correct shape to pass (**Figure 5**), an environment with characteristic functional filters acts as a sorter of functions. This sorter, which is determined by the environment (niche theory) and which is a characteristic of that environment type, is used to select microbes from nearby pools. Similar to the game in which right-shaped blocks, despite their color, have equal possibility of passing the shape filter and getting into the sorter, microbes with genes that match the environmental functional filters, irrespective of their taxa, can be selected stochastically (neutral theory). Next, these randomly selected successful microbes outcompete other microbes, colonize the environment, and occupy the niche. Therefore, different microbial species with similar properties or functions can randomly occupy the same niche in an ecosystem. Like oil reservoirs, soil, freshwater, marine, and extreme environments have their specific environmental factors and form four types of sorters with environment-specific functional filters for selecting genes.

Although the shape sorter equally allows the right-shaped blocks with different colors to pass through, colors with more number of blocks have higher possibilities to get into the shape sorter. Similarly, although the functional sorter is environment determined and is relatively universal within an environment type, microbes close to the sorter may be endemic because of their biogeographic patterns, especially at a larger scale (Martiny et al., 2006). Therefore, endemic differences in microbial composition should play a role in the assembly of microbial communities by offering endemic pools of diverse microbial candidates for selection. Thus, the shape-sorting system is comprehensively determined by the niche and neutral theories, local legacies of historical microbial events on a large scale, and differences among different microbial taxa. For example, crude oil has its own functional sorting system that selects specific functions from different taxa. However, further research is needed to test this hypothesis.

In summary, we characterized microbial functional gene compositions in crude oil from two geographically distant oil reservoirs (QH and DQ) in China and found that these genes clustered with genes from extreme environments but not with genes from soil, freshwater, and marine environments. Although microbial compositions were taxonomically different between oil reservoirs with different geochemical conditions and between different wells from the same oilfield with similar geochemical conditions, functional gene compositions in the metagenomes of these oil reservoirs showed high similarities. The preserved common complete methanogenic hydrocarbon degradation pathways and enriched stress response genes were the key factors for distinguishing the metagenomes of oil reservoirs and extreme environments from those of soil, freshwater, and marine environments. These results suggested that microbial communities in oil reservoirs with consistent functional profiles are functionally selected by the niche theory but taxonomically selected by the neutral theory. Moreover, all the environmental metagenomes were clustered according to their isolation environments based on the hierarchical clustering of functional genes, suggesting that both selective and neutral processes were involved in the assembly of microbial communities in oil reservoirs.

## Author Contributions

YN and J-YZ contributed equally to this work. YN, X-LW, and FZ designed the research. YN, J-YZ, and Y-QT performed research. J-YZ re-analyzed the datasets, and revised the manuscript. PG and YY contributed analytic tools. YN, J-YZ, YY, X-LW, and FZ analyzed data. YN, J-YZ, YY, and X-LW wrote the paper.

## Conflict of Interest Statement

The authors declare that the research was conducted in the absence of any commercial or financial relationships that could be construed as a potential conflict of interest.
