# Supplementary material for: Species Divergence vs. Functional Convergence Characterizes Crude Oil Microbial Community Assembly
Source: Front Microbiol. 2016 Aug 12;7:1254. doi: 10.3389/fmicb.2016.01254 (PMC4981601; doi:10.3389/fmicb.2016.01254)
Supplement: Supplementary file 1 [file Data_Sheet_1.PDF]

Supplementary Material

Species divergence vs functional convergence characterizes crude oil microbial community assembly

Yong Nie†, Jie-Yu Zhao†, Yue-Qin Tang, Peng Guo, Yunfeng Yang, Xiao-Lei Wu*, Fangqing Zhao*

†These authors contributed equally to this work.

*** Correspondence:** Xiao-Lei Wu: xiaolei_wu@pku.edu.cn; Fangqing Zhao: zhfq@mail.biols.ac.cn

1 Supplementary Figures and Tables

1.1 Supplementary Figures

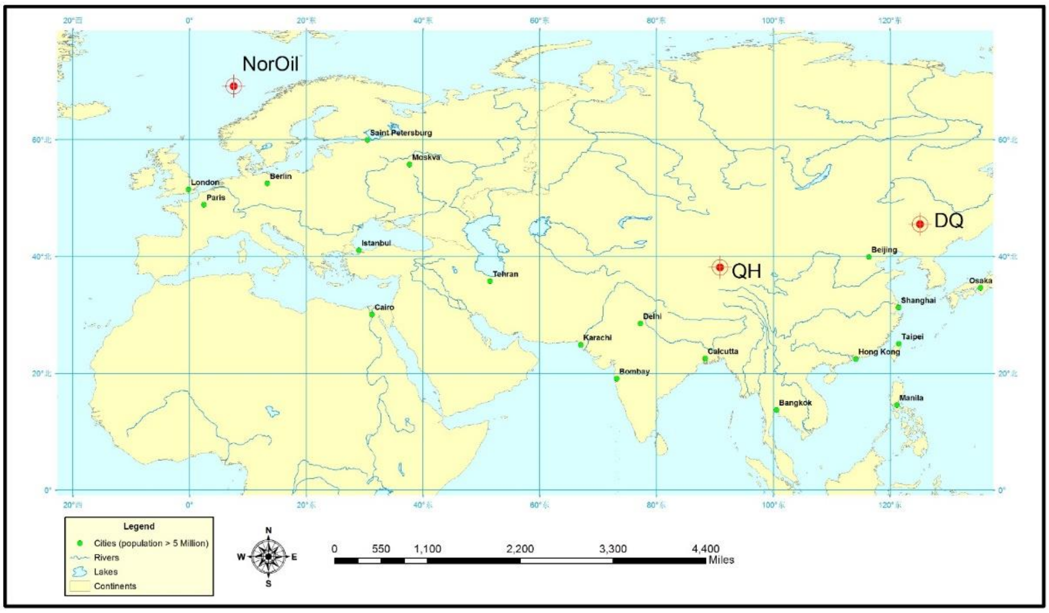

Figure S1. Geographical locations of QH, DQ and NorOil. The map was created with ArcGIS 10.2

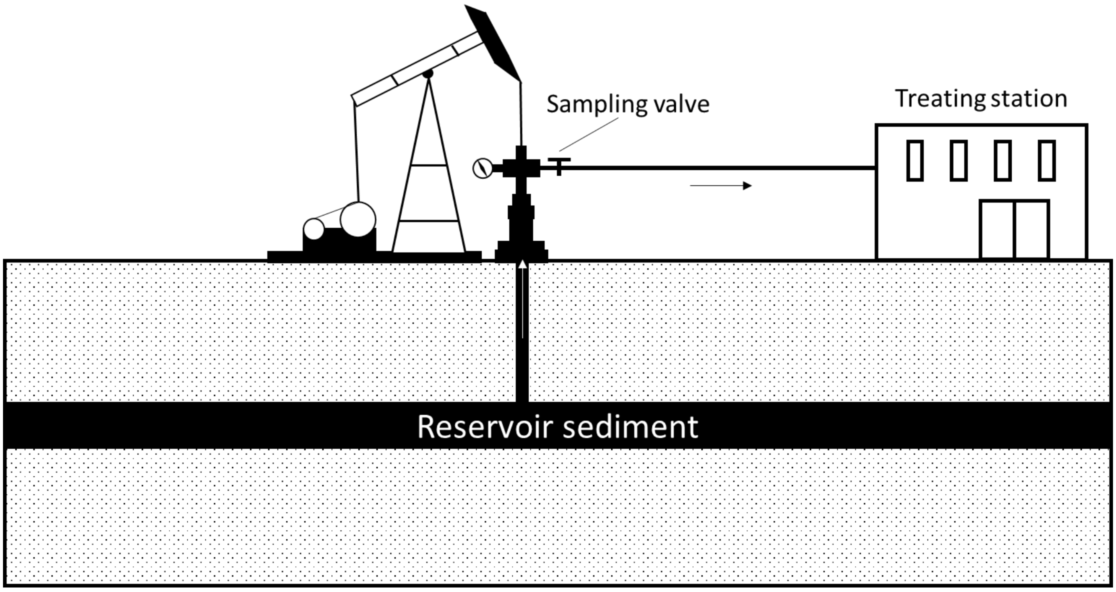

Figure S2. Schematic representation of the procedure for sample collection

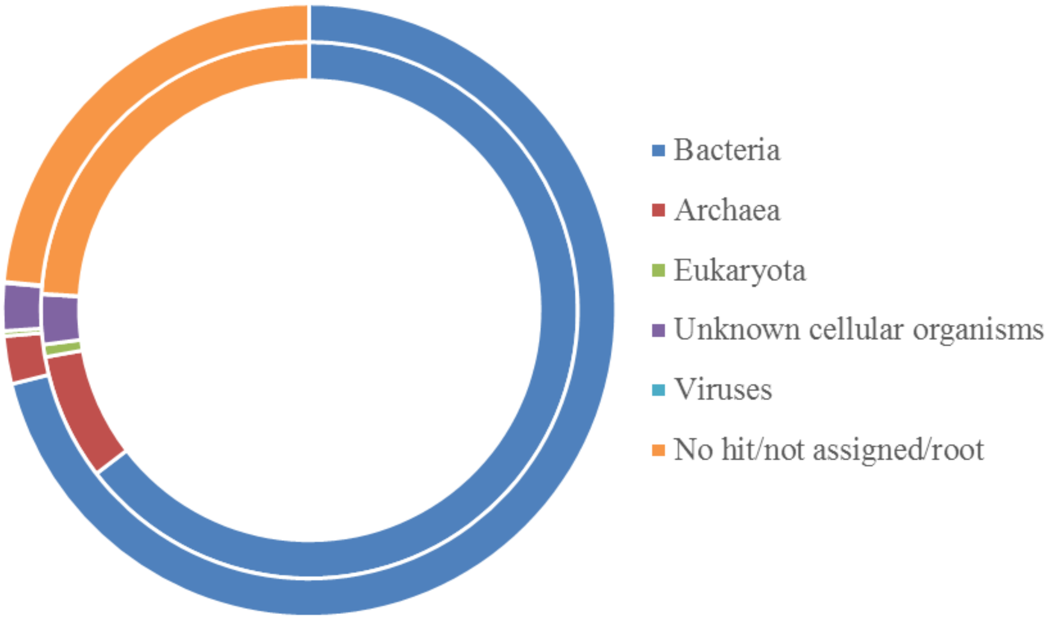

Figure S3. Comparison of reads assigned distributions in QH and DQ. Inner circle, QH; outer circle, DQ.

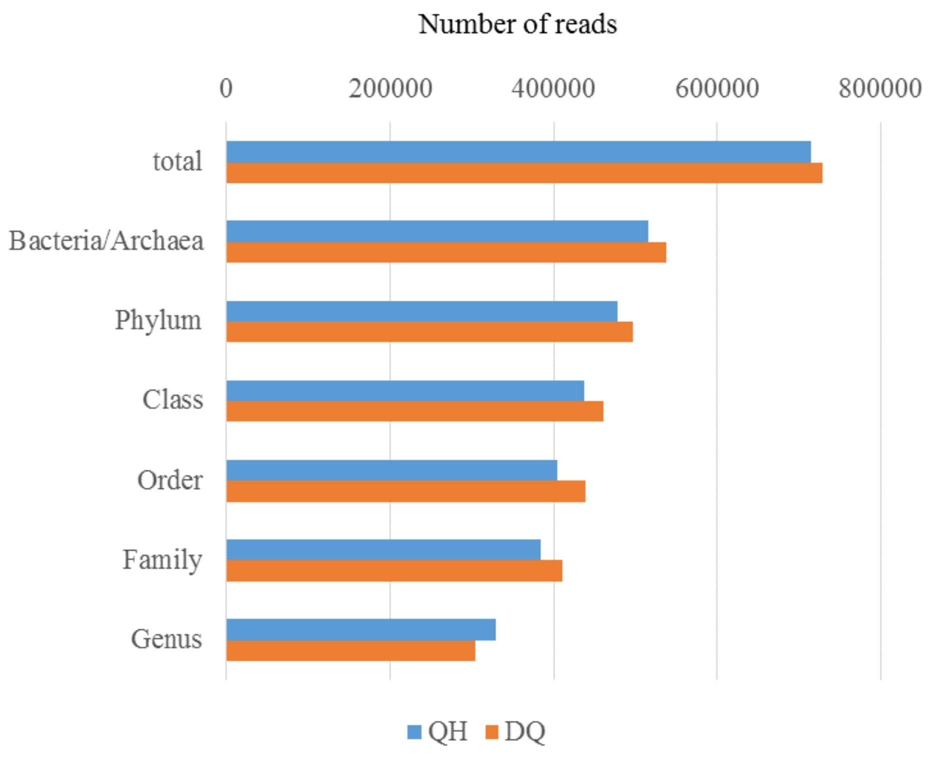

Figure S4. Number of reads in QH and DQ assigned to different taxonomic levels

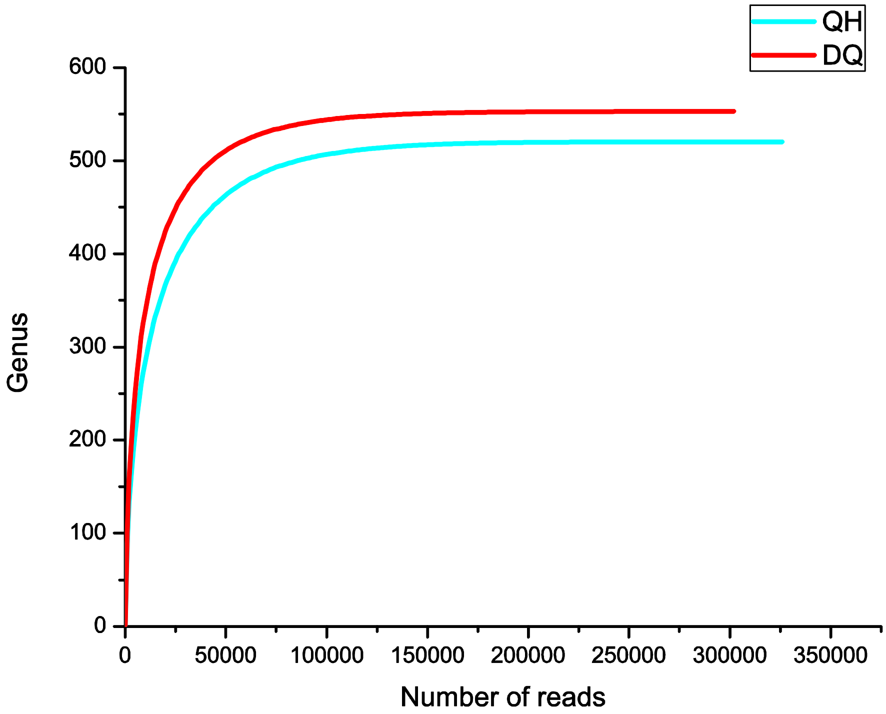

Figure S5. Rarefaction plot of the merged long reads based on the taxonomic information at the genus level retrieved from the annotation results against NR using BLASTX.

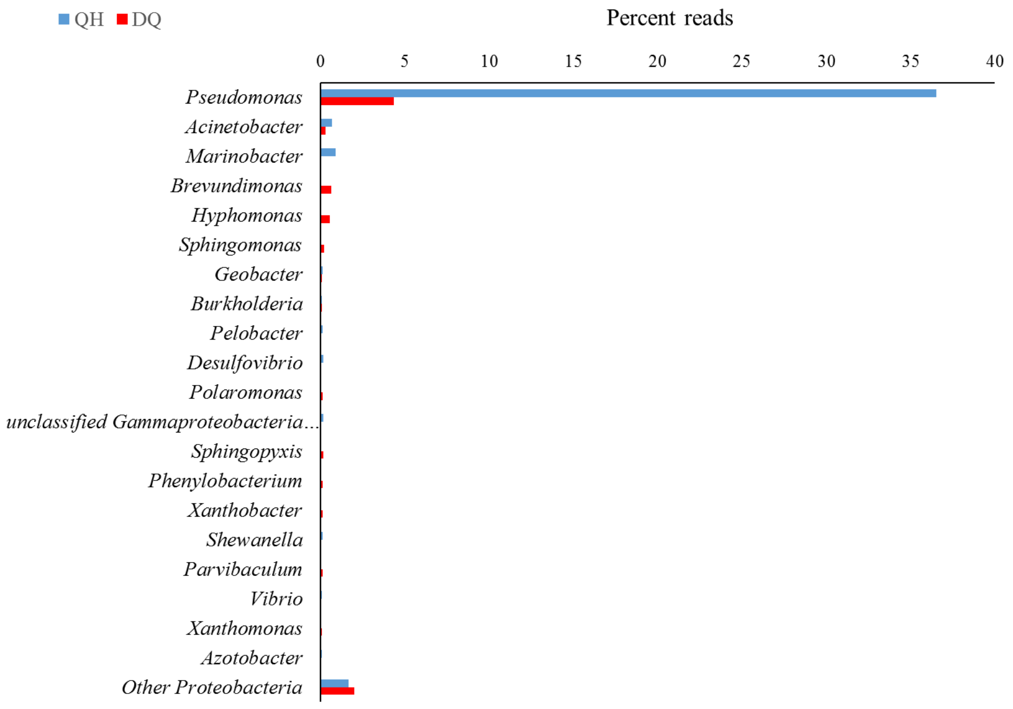

Figure S6. Comparison of the composition of *Proteobacteria* at the genus level in QH and DQ

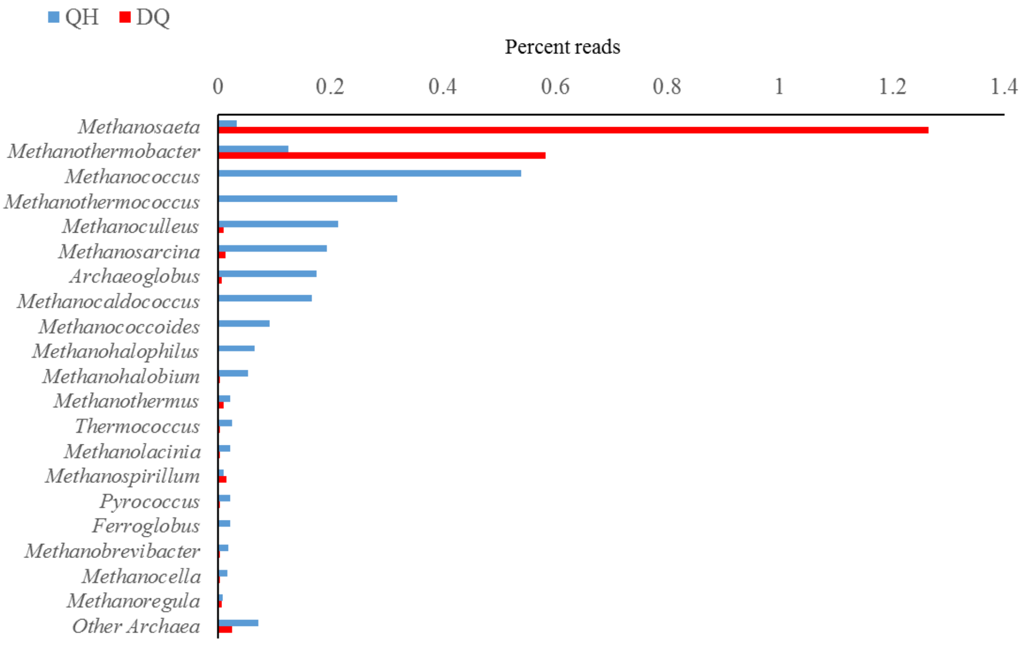

Figure S7. Comparison of the archaeal composition at the genus level in QH and DQ. The top 20 abundant genera in QH and DQ were shown, and the remaining genera were combined together as “Other archaea”.

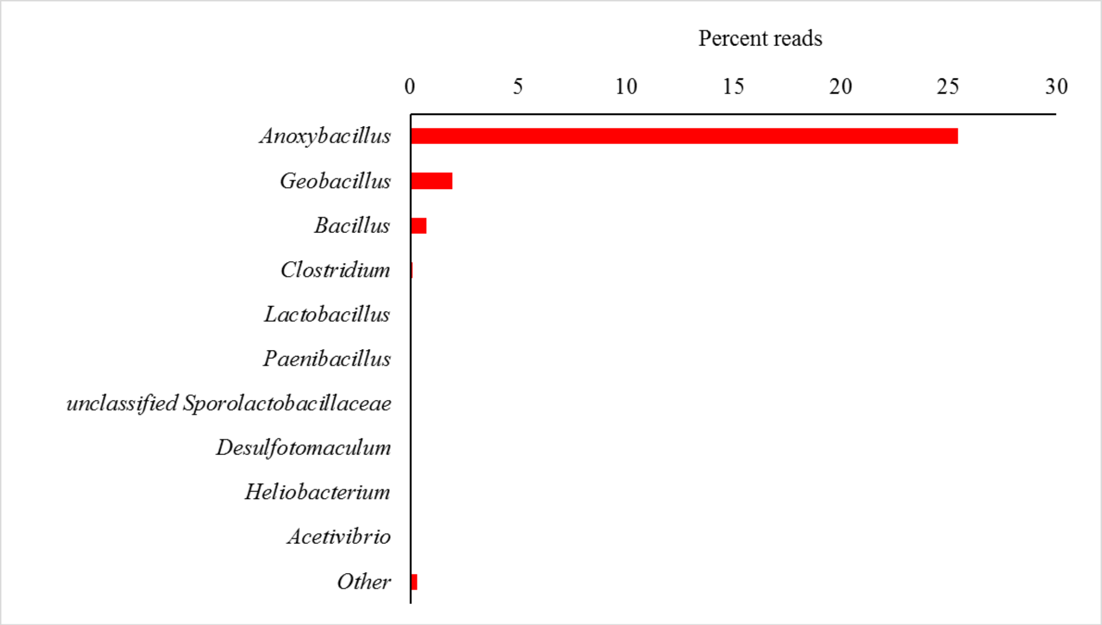

Figure S8. The composition of *Firmicutes* in DQ at the genus level.

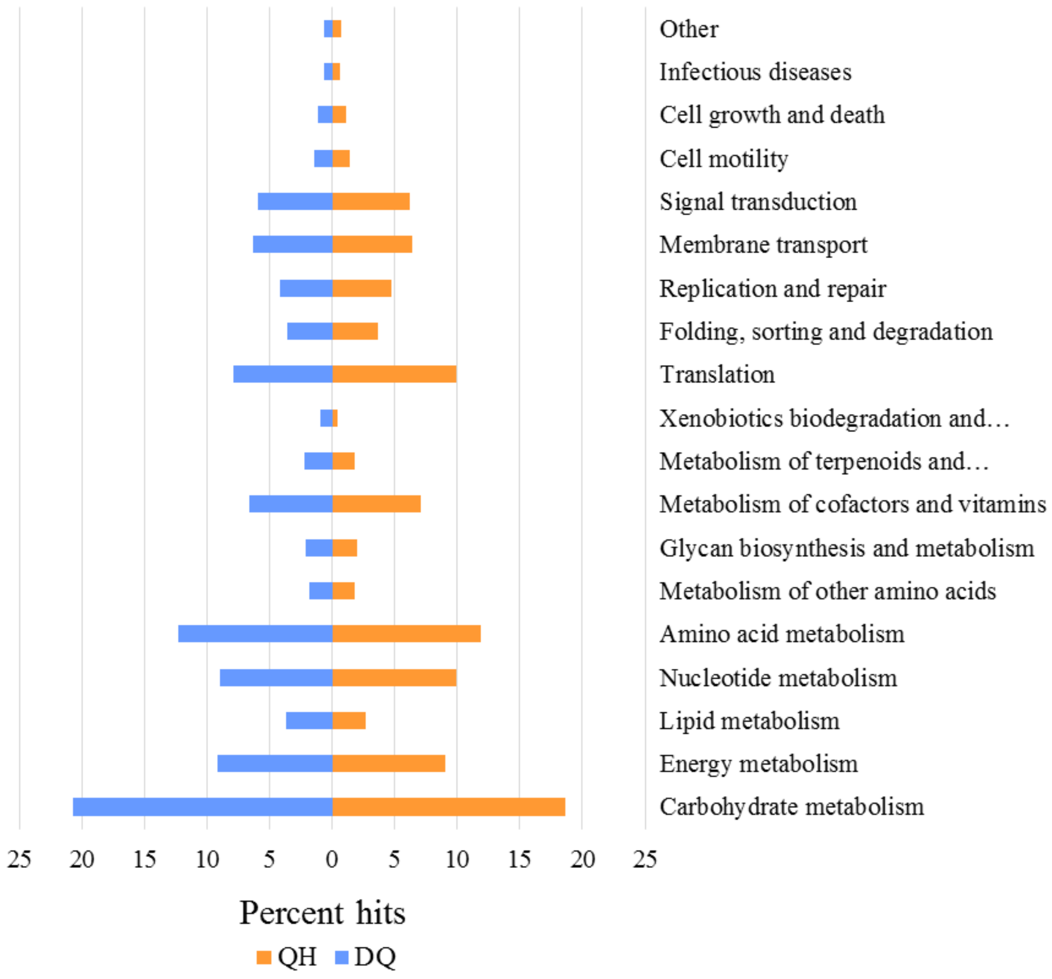

Figure S9. The functional composition of crude oil metagenomes in KEGG database. The ORFs of assembled contigs were predicted using MetaGeneMark program and then compared against the KEGG Orthologous (KOs) database respectively to assess the functional classification.

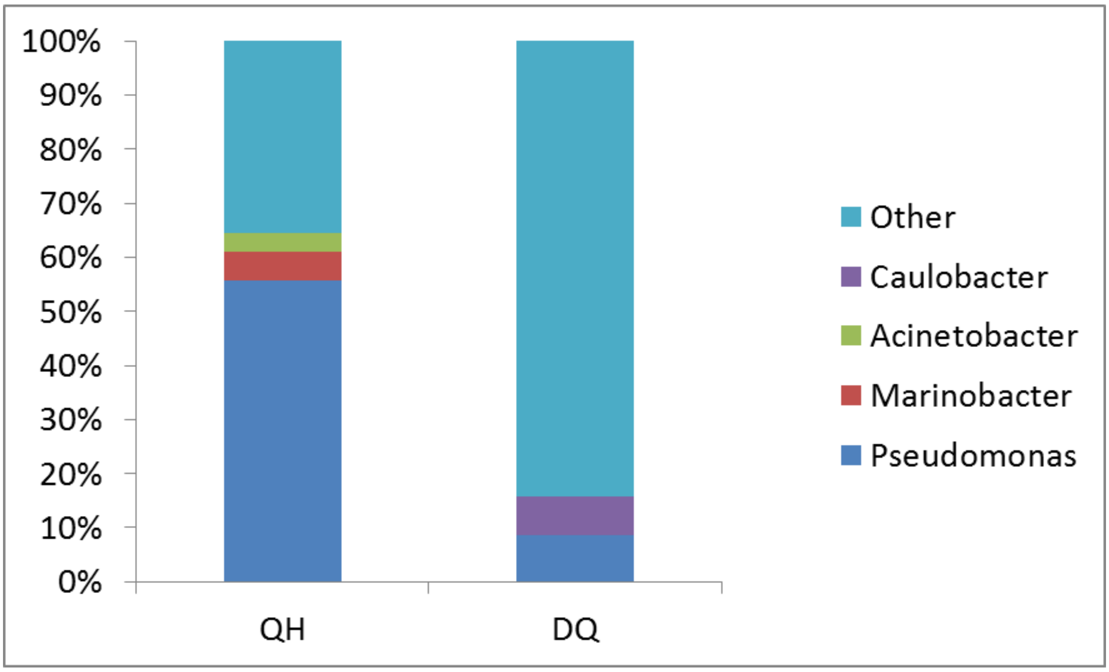

Figure S10. Taxonomic assignment of *alkB* genes in both QH and DQ

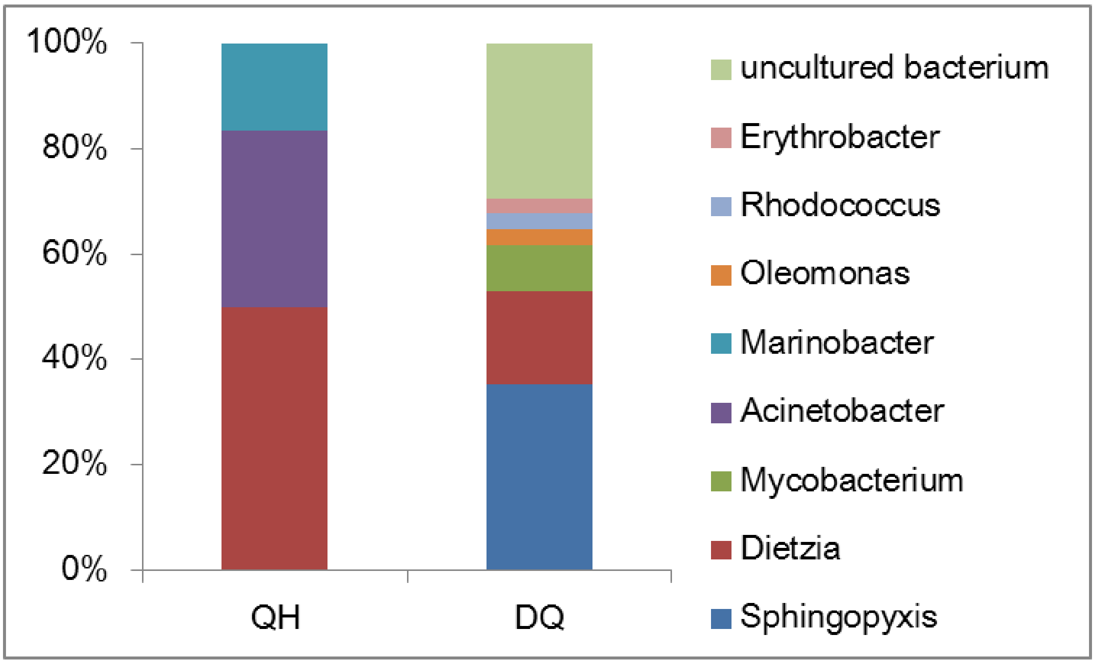

Figure S11. Taxonomic assignment of CYP153 genes in both QH and DQ.

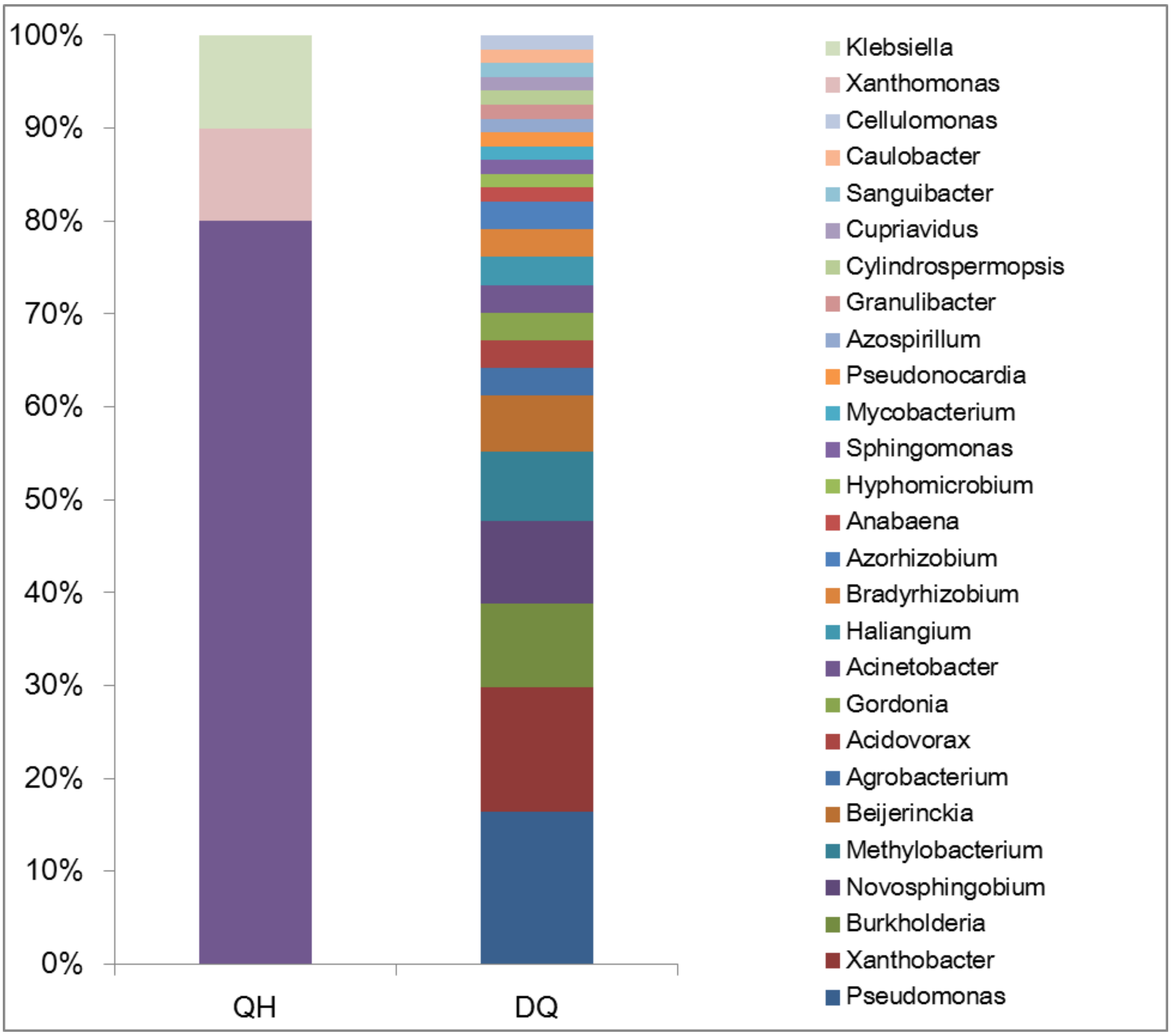

Figure S12. Taxonomic assignment of *ladA* genes in both QH and DQ.

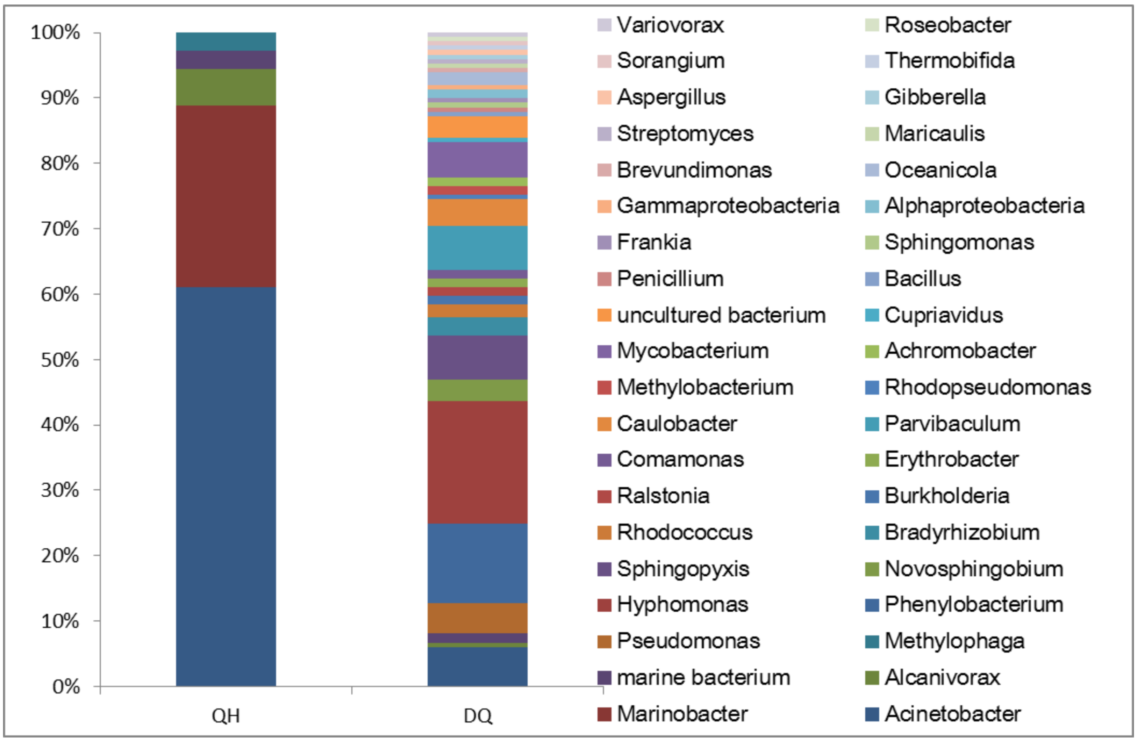

Figure S13. Taxonomic assignment of *almA* genes in both QH and DQ

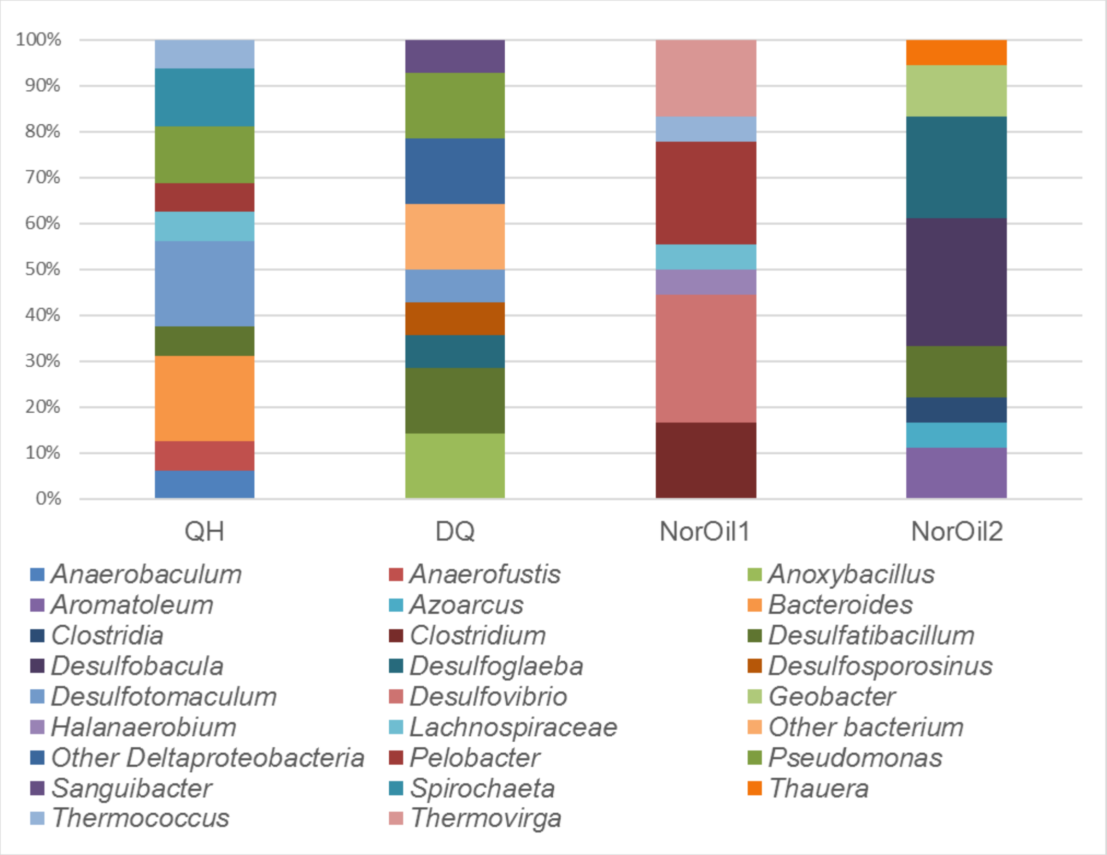

Figure S14. Taxonomic assignment of *assA/bssA* genes in QH, DQ and NorOil metagenomes

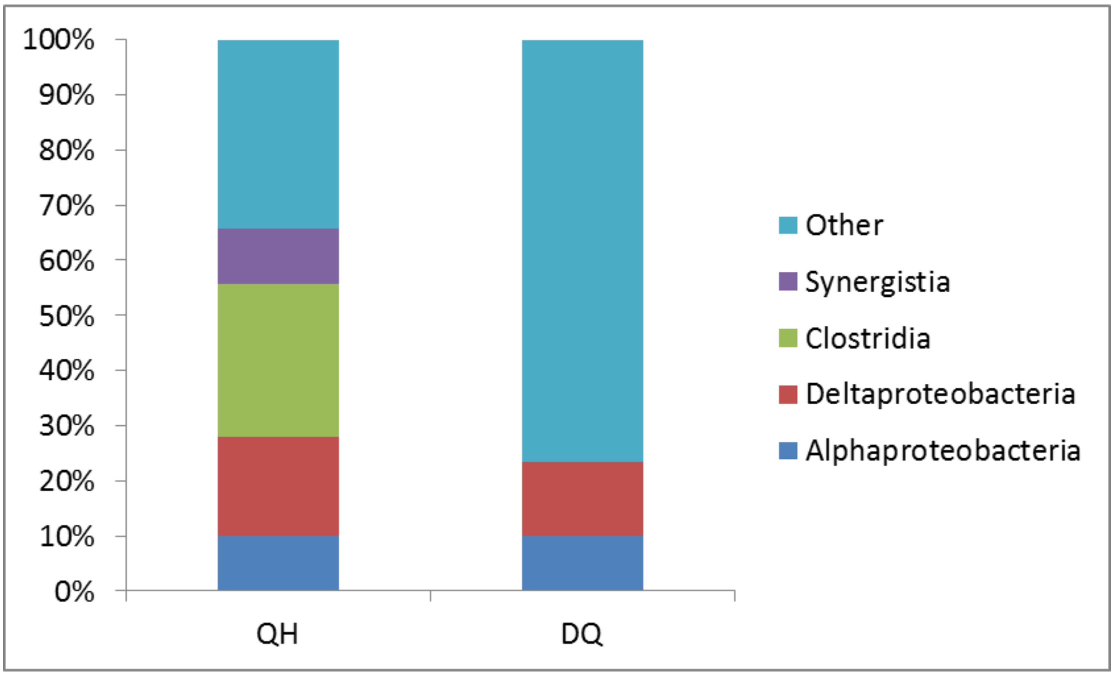

Figure S15. Taxonomic assignment of CODH/ACS genes in both QH and DQ

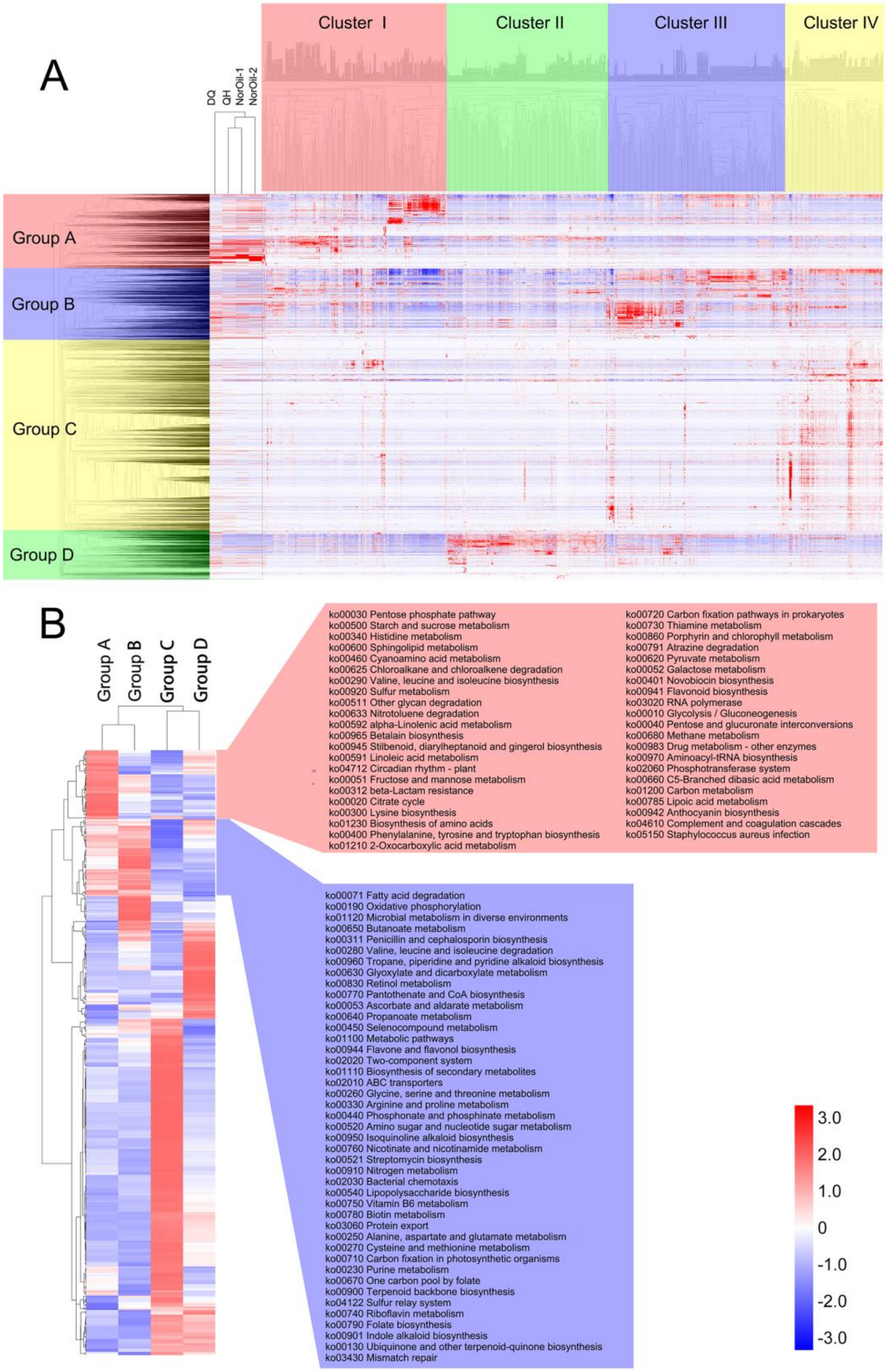

Figure S16. Hierarchical clustering analysis of KEGG genes in both the QH and DQ metagenomes together with the reference metagenomes. (A) All the samples were clustered into four groups based on their isolation environments. All the genes were enriched into four major groups. In all, 2,984, 1,701, 2,558, and 7,339 enriched KOs were hot in groups A, B, C, and D, respectively (Table S10 and S11). (B) Genes in the four groups were affiliated to the KEGG pathways and were clustered.

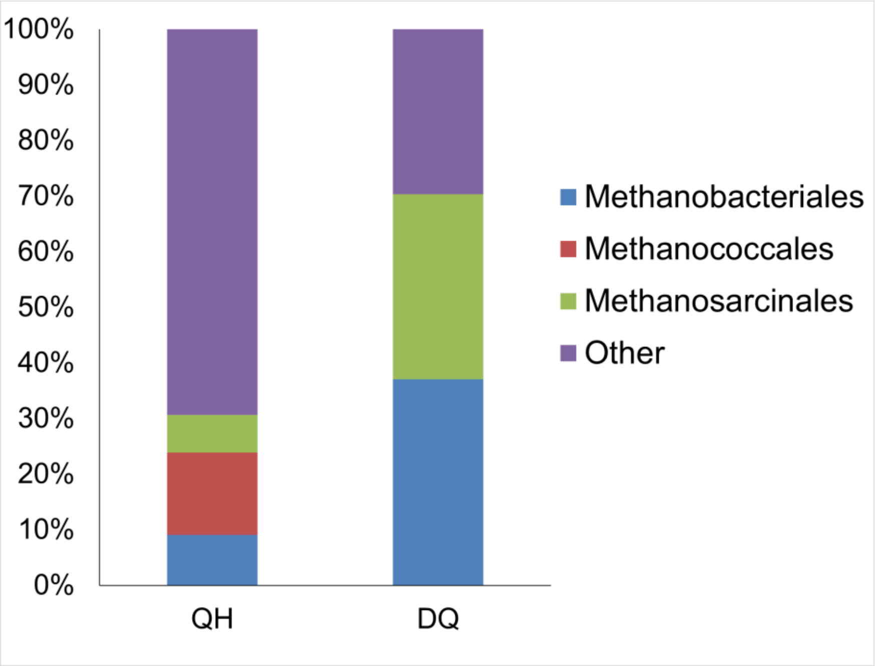

Figure S17. Taxonomic assignment of *mcrA* genes in both QH and DQ.

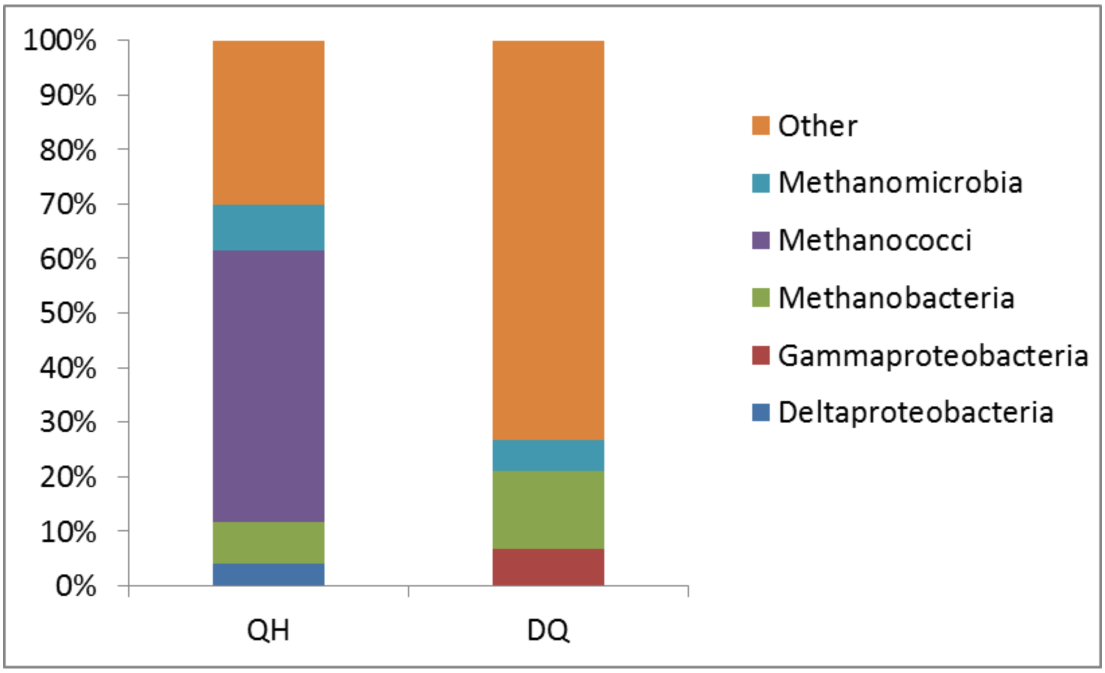

Figure S18. Taxonomic assignment of *nif* genes in both QH and DQ

1.2 Supplementary Tables

Table S1 Reference sequences used in this work

	Accession ID
BssA/AssA	AAC38454.1, CAA05052.1, AAK50372.1, AAM34597.1, YP_158060.1, BAC05501.1, ABK15654.1, BAF63031.1, ABM92934.1, ABM92936.1, ABM92937.1, ABM92938.1, ABM92939.1, CAO72221.1, CBH30982.1, ACM20791.1, ACM20801.1, ABO30979.1, ABO30980.1, ADJ93876.1, CAO72219.1, CAO72220.1, CAO72222.1, CCK78655.1, CCK79722.1, ABH11460.1, ABH11461.1, ADJ51097.1, CBK27727.1, CAO03074.1
LadA	YP_001127577.1, ABV66256.1, YP_003987695.1, YP_004586379.1, ZP_02358289.1, AEN91250.1, YP_233462.1, YP_002801477.1, YP_235943.1, YP_888664.1, YP_003778123.1, YP_003732559.1
AlmA	ABQ18228.1, ABQ18224.1, ZP_03824363.1, YP_587356.1, YP_002768846.1, YP_961086.1, YP_692002.1, ZP_01913854.1, YP_001086053.1, YP_047698.1, AEM75111.1, ZP_08438244.1

Tables S2 Microbial compositions at the phylum level in QH and DQ

Phylum	Abundance	
	QH	DQ
Proteobacteria	80.83%	26.76%
Euryarchaeota	11.14%	3.64%
Firmicutes	3.75%	64.25%
Deferribacteres	1.18%	0.20%
Synergistetes	0.72%	0.01%
Thermotogae	0.70%	0.05%
Bacteroidetes	0.54%	1.44%
Actinobacteria	0.29%	2.30%
Chloroflexi	0.24%	0.20%
candidate division WWE1	0.11%	0.01%
Cyanobacteria	0.10%	0.11%
Spirochaetes	0.06%	0.03%
Chlorobi	0.05%	0.04%
Aquificae	0.04%	0.02%
Planctomycetes	0.04%	0.12%
Fusobacteria	0.03%	0.01%
Deinococcus-Thermus	0.03%	0.08%
Crenarchaeota	0.03%	0.01%
Tenericutes	0.02%	0.01%
Verrucomicrobia	0.02%	0.43%
Nitrospirae	0.01%	0.03%
Chlamydiae	0.01%	<0.01%
Chrysiogenetes	0.01%	0.01%
Acidobacteria	0.01%	0.12%
candidate division TM7	0.01%	0.04%
Dictyoglomi	0.01%	0.01%
Gemmatimonadetes	0.01%	0.02%
Lentisphaerae	0.01%	0.01%
Thermobaculum	<0.01%	<0.01%
candidate division NC10	<0.01%	0.01%
Korarchaeota	<0.01%	<0.01%
Thaumarchaeota	<0.01%	0.01%
Nanoarchaeota	<0.01%	Not detected
Elusimicrobia	<0.01%	<0.01%
Fibrobacteres	Not detected	<0.01%
Poribacteria	Not detected	<0.01%

Table S3 Archaeal compositions at the genus level in QH and DQ

Genus	Abundance	
	QH	DQ
Methanosaeta	0.03%	1.27%
Methanothermobacter	0.13%	0.58%
Methanococcus	0.54%	<0.01%
Methanothermococcus	0.32%	<0.01%
Methanoculleus	0.21%	0.01%
Methanosarcina	0.19%	0.01%
Archaeoglobus	0.18%	0.01%
Methanocaldococcus	0.17%	<0.01%
Methanococcoides	0.09%	<0.01%
Methanohalophilus	0.07%	<0.01%
Methanohalobium	0.05%	<0.01%
Methanothermus	0.02%	0.01%
Thermococcus	0.03%	<0.01%
Methanolacinia	0.02%	<0.01%
Methanospirillum	0.01%	0.01%
Pyrococcus	0.02%	<0.01%
Ferroglobus	0.02%	<0.01%
Methanobrevibacter	0.02%	<0.01%
Methanocella	0.02%	<0.01%
Methanoregula	0.01%	0.01%
Methanosphaerula	0.01%	<0.01%
Ferroplasma	0.01%	Not detected
Aciduliprofundum	0.01%	Not detected
Sulfolobus	<0.01%	<0.01%
Methanocorpusculum	<0.01%	<0.01%
Methanopyrus	<0.01%	Not detected
Methanosphaera	<0.01%	<0.01%
Ignisphaera	<0.01%	Not detected
Candidatus Parvarchaeum	<0.01%	Not detected
Natronomonas	<0.01%	<0.01%
Methanobacterium	<0.01%	<0.01%
Candidatus Korarchaeum	<0.01%	<0.01%
Halorhabdus	<0.01%	<0.01%
Nitrosopumilus	<0.01%	<0.01%
Thermofilum	<0.01%	Not detected
Methanotorris	<0.01%	Not detected
Halorubrum	<0.01%	Not detected
Nanoarchaeum	<0.01%	Not detected
Staphylothermus	<0.01%	Not detected
Halobacterium	<0.01%	Not detected
Thermoplasma	<0.01%	Not detected
Pyrobaculum	<0.01%	Not detected
Haladaptatus	<0.01%	Not detected

Methanolobus	<0.01%	Not detected
Halalkalicoccus	<0.01%	Not detected
Cenarchaeum	Not detected	<0.01%
Haloferax	<0.01%	Not detected
Haloterrigena	<0.01%	Not detected
Nitrososphaera	Not detected	<0.01%

Table S4 Number of genes found in QH and DQ for biosynthesis of glycolipids.

Genes in biosynthesis of glycolipids	QH	DQ
acyltransferase	413	752
rhamnosyltransferase	0	5
mannosyltransferase	26	50
glucosyltransferase	14	91
heptosyltransferase	26	66
galactosyltransferase	5	6

Table S5 Number of genes found in QH and DQ for biosynthesis of lipopeptides

Genes in biosynthesis of lipopeptides	QH	DQ
surfactin	0	1
gramicidin	1	5
arthrofactin	0	0
Iturin A	0	2
gramicidin	1	5

Table S6 Representative samples in Cluster I

Genome name/Sample name	IMG Genome ID /NCBI SRA ID
QH	PRJNA251580
DQ	PRJNA251580
NorOil1	Kotlar et al., 2011, *Environ. Microbiol. Rep.* 3**,** 674-681.
NorOil2	Lewin et al., 2014, *Environ. Microbiol.* 16**,** 545-558.
Acid Mine Drainage	3300003083
Baltic Sea site KBA sample	3300000134
Bath Hot Springs	2007309001
Beowulf Spring	3300000346
Cistern Spring	3300000398
Conch Spring	3300000397
Crude oil metagenome 2	3300001685
Deep Marine Sediments	3300001854
Echinus Geyser	3300003708
Elkhorn Slough mat	3300000353
Grendel Spring	3300000348
Groundwater microbial communities from subsurface biofilms in sulfidic aquifier in Frasassi Gorge, Italy	3300000230
Guerrero Negro salt ponds hypersaline mat	2004247001
Hot Lake mat	3300001112
Hot spring microbial communities from Yellowstone National Park	2022920004
Hypersaline microbial communities from Antarctic Deep Lake	2084038011
Hypersaline water microbial communities from Lake Tyrrell, Victoria, Australia	2124908030
King George Island	3300000129
Lentic microbial communities from Lake Waban, Wellesley MA	3300000497
Mammoth Hot Spring	3300003076
Marine sediment microbial communities from Kolumbo Volcano mats, Greece	3300002242
Microbial Communities from Little Sippewissett Salt Marsh, Woods Hole	3300000499
Monarch Geyser	3300001309
NGEE Surface sample	3300001454
Octopus Spring Streamers	3300000345
Saline water microbial communities from Great Salt Lake, Utah	2058419001
Sediment microbial communities from Arctic Ocean, off the coast from Alaska	2100351012

Sediment microbial communities from Great Boiling Spring, Nevada	3300000109
Sediment microbial communities from Lake Washington, Seattle	2088090007
Sediment microbial community from Etoliko Lagoon, Greece	3300002466
Sinkhole freshwater microbial communities from Lake Huron, US	3300005701
Soda Lake	3300000576
Soil microbial communities sample from Light Crust, Colorado Plateau, Green Butte	3300000095
Subsurface groundwater monitoring well	3300001380
Washburn Spring	2022920019
Water microbial communities from Great Boiling Spring, Nevada	2619619099
Wetland microbial communities from Twitchell Island in the Sacramento Delta	3300000100

Table S7 Representative samples in Cluster II

Genome name/Sample name	IMG Genome ID
Amazon Forest 2010	3300000816
Arabidopsis rhizosphere microbial communities from University of North Carolina	3300000652
Black Spruce, Ontario combined	3300001593
Forest Soil microbial communities	3300001621
Hopland Soil Metagenome 1 Hopland Soil	3300001305
Jack Pine, Ontario site 1_JW_OM1H0_M1, ASSEMBLY_DATE=20130226)	3300001160
Kansas native prairie amended with acetate	3300000754
Luquillo Experimental Forest Soil, Puerto Rico	3300000701
Maize field bulk soil microbial communities from University of Illinois Energy Farm, Urbana, IL Bulk soil sample from field growing	2044078000
Mediterranean Blodgett	3300001147
Microbial Forest Soil	3300001639
Miscanthus field bulk soil microbial communities from University of Illinois Energy Farm, Urbana	2044078003
Miscanthus rhizosphere microbial communities from Kellogg Biological Station, MSU	3300005271
NGEE Surface sample	3300001454
Peat Soil Metagenome	3300001356
Replicate Kansas native prairie amended with acetate	3300000519
Rhopaloeides odorabile metagenome	3300000389
Soil microbial communities from Great Prairies, sample from Iowa	3300000364
Soil microbial communities from Great Prairies, sample from Kansas Corn soil Kansas Corn	2067725004
Soil microbial communities from Minnesota Farm	2001200001
Soil microbial communities from permafrost in Bonanza Creek, Alaska	2124908036
Soil microbial communities from sample at FACE Site	2124908009
Soil microbial communities sample from Dark Crust, Colorado Plateau, Green Butte Dark	2209111000
Switchgrass field bulk soil microbial communities from University of Illinois Energy Farm, Urbana	2044078005
Switchgrass rhizosphere microbial community from Michigan, US	3300005280
Texas A ecozone	3300001461

Table S8 Representative samples in Cluster III

Genome name/Sample name	IMG Genome ID
Air microbial communities Singapore indoor air filters	2003000006
Mangrove A Sediment - Bioluminescent Bay in La Paraguera,PR	3300000888
Deep seawater metagenome	3300001582
Estuarine microbial communities from Columbia River	2236876004
Fossil microbial community from Whale Fall at Santa Cruz Basin of the Pacific Ocean	2001200004
Hydrothermal vent microbial communities from Guaymas and Carmen Basins, Gulf of California	2061766003
Lake Vida Brine Hole Two >0.2 micron fraction	3300000405
Line P August 2008 P12 1000m	3300000322
Marine Bacterioplankton communities from Antarctic	3300005372
Marine microbial communities from Columbia River	2236876002
Marine microbial communities from Deepwater Horizon Oil Spill	2081372001
Marine microbial communities from Delaware Coast, sample from Delaware	3300000368
Marine microbial communities from the Eastern Subtropical North Pacific Ocean	2537562245
Marine planktonic communities from Hawaii Ocean	2014642002
Marine sediment microbial communities from Kolumbo Volcano mats, Greece	3300002242
Marine Trichodesmium cyanobacterial communities from the Bermuda Atlantic	2156126005
Marine Trichodesmium cyanobacterial communities from the North Pacific Subtropical Gyre outside Oahu	3300005722
Methylotrophic community from Lake Washington sediment Formate enrichment	2006207004
Microbial Communities from Little Sippewissett Salt Marsh	3300000499
Miscanthus rhizosphere microbial communities from Kellogg Biological Station, MSU	3300005338
Naphthalene biodegradation metagenome	3300001299
Oak Ridge Pristine Groundwater	3300000178
Saanich Inlet	3300003601
Saline water microbial communities from Great Salt Lake, Utah	2058419001
Soda Lake	3300000576
Subsurface groundwater monitoring well GMW36 contaminated	3300001338
Tierra del Fuego site	3300000122
Uranium Contaminated Groundwater FW106	2007427000

Table S9 Representative samples in Cluster IV

Genome name/Sample name	IMG Genome ID
Fresh water microbial communities from LaBonte Lake	2166559021
Freshwater microbial communities from Lake Mendota, WI	3300002368
Freshwater microbial communities from Trout Bog Lake, WI	2199352002
Hot Lake mat section	3300001112
Lake Erie	3300000756
Lake Mendota	3300002296
Lotic microbial communities from Mississippi Rive	3300000206
Marine microbial communities from Delaware Coast	3300000368
Sediment microbial communities from Lake Washington	2088090007
Soil microbial communities from permafrost in Bonanza Creek, Alaska	2124908036
Switchgrass rhizosphere microbial community from Michigan, US	3300005280

Table S10 Pathways with high abundance in Group A

**Pathways specific in Group A**
ko00030 Pentose phosphate pathway
ko00500 Starch and sucrose metabolism
ko00340 Histidine metabolism
ko00600 Sphingolipid metabolism
ko00460 Cyanoamino acid metabolism
ko00625 Chloroalkane and chloroalkene degradation
ko00290 Valine, leucine and isoleucine biosynthesis
ko00920 Sulfur metabolism
ko00511 Other glycan degradation
ko00633 Nitrotoluene degradation
ko00592 alpha-Linolenic acid metabolism
ko00965 Betalain biosynthesis
ko00945 Stilbenoid, diarylheptanoid and gingerol biosynthesis
ko00591 Linoleic acid metabolism
ko04712 Circadian rhythm - plant
ko00051 Fructose and mannose metabolism
ko00312 beta-Lactam resistance
ko00020 Citrate cycle
ko00300 Lysine biosynthesis
ko01230 Biosynthesis of amino acids
ko00400 Phenylalanine, tyrosine and tryptophan biosynthesis
ko01210 2-Oxocarboxylic acid metabolism
ko00720 Carbon fixation pathways in prokaryotes
ko00730 Thiamine metabolism
ko00860 Porphyrin and chlorophyll metabolism
ko00791 Atrazine degradation
ko00620 Pyruvate metabolism
ko00052 Galactose metabolism
ko00401 Novobiocin biosynthesis
ko00941 Flavonoid biosynthesis
ko03020 RNA polymerase
ko00010 Glycolysis / Gluconeogenesis
ko00040 Pentose and glucuronate interconversions
ko00680 Methane metabolism
ko00983 Drug metabolism - other enzymes
ko00970 Aminoacyl-tRNA biosynthesis
ko02060 Phosphotransferase system
ko00660 C5-Branched dibasic acid metabolism
ko01200 Carbon metabolism
ko00785 Lipoic acid metabolism
ko00942 Anthocyanin biosynthesis
ko04610 Complement and coagulation cascades
ko05150 Staphylococcus aureus infection
**Pathways with high abundance both in Group A and Group B**
ko00071 Fatty acid degradation
ko00190 Oxidative phosphorylation
ko01120 Microbial metabolism in diverse environments

ko00650 Butanoate metabolism
ko00311 Penicillin and cephalosporin biosynthesis
ko00280 Valine, leucine and isoleucine degradation
ko00960 Tropane, piperidine and pyridine alkaloid biosynthesis
ko00630 Glyoxylate and dicarboxylate metabolism
ko00830 Retinol metabolism
ko00770 Pantothenate and CoA biosynthesis
ko00053 Ascorbate and aldarate metabolism
ko00640 Propanoate metabolism
ko00450 Selenocompound metabolism
ko01100 Metabolic pathways
ko00944 Flavone and flavonol biosynthesis
ko02020 Two-component system
ko01110 Biosynthesis of secondary metabolites
ko02010 ABC transporters
ko00260 Glycine, serine and threonine metabolism
ko00330 Arginine and proline metabolism
ko00440 Phosphonate and phosphinate metabolism
ko00520 Amino sugar and nucleotide sugar metabolism
ko00950 Isoquinoline alkaloid biosynthesis
ko00760 Nicotinate and nicotinamide metabolism
ko00521 Streptomycin biosynthesis
ko00910 Nitrogen metabolism
ko02030 Bacterial chemotaxis
ko00540 Lipopolysaccharide biosynthesis
ko00750 Vitamin B6 metabolism
ko00780 Biotin metabolism
ko03060 Protein export
ko00250 Alanine, aspartate and glutamate metabolism
ko00270 Cysteine and methionine metabolism
ko00710 Carbon fixation in photosynthetic organisms
ko00230 Purine metabolism
ko00670 One carbon pool by folate
ko00900 Terpenoid backbone biosynthesis
ko04122 Sulfur relay system
ko00740 Riboflavin metabolism
ko00790 Folate biosynthesis
ko00901 Indole alkaloid biosynthesis
ko00130 Ubiquinone and other terpenoid-quinone biosynthesis
ko03430 Mismatch repair

Table S11 Number of KOs distributed in different pathways in different Groups

Enriched pathways	Number of KOs (abundance of all enriched KOs) in this pathway			
	Group A	Group B	Group C	Group D
ko01200 Carbon metabolism	146(0.051)	63(0.0229)	40(0.0055)	38(0.0206)
ko01230 Biosynthesis of amino acids	110(0.039)	58(0.0211)	32(0.0044)	18(0.0098)
ko00680 Methane metabolism	93(0.0326)	24(0.0087)	14(0.0019)	20(0.0108)
ko00720 Carbon fixation pathways in prokaryotes	55(0.0193)	25(0.0091)	5(0.0007)	10(0.0054)
ko02060 Phosphotransferase system	53(0.0186)	11(0.0040)	6(0.0008)	4(0.0022)
ko00010 Glycolysis / Gluconeogenesis	46(0.0161)	16(0.0058)	16(0.0022)	10(0.0054)
ko00620 Pyruvate metabolism	41(0.0144)	25(0.0091)	5(0.0007)	13(0.0070)
ko00051 Fructose and mannose metabolism	38(0.0133)	21(0.0076)	11(0.0015)	9(0.0049)
ko00500 Starch and sucrose metabolism	36(0.0126)	18(0.0066)	24(0.0033)	17(0.0092)
ko00052 Galactose metabolism	33(0.0116)	11(0.0040)	19(0.0026)	3(0.0016)
ko00860 Porphyrin and chlorophyll metabolism	32(0.0112)	20(0.0073)	41(0.0056)	9(0.0049)
ko00970 Aminoacyl-tRNA biosynthesis	31(0.0109)	6(0.0022)	4(0.0005)	2(0.0011)
ko01210 2-Oxocarboxylic acid metabolism	30(0.0105)	10(0.0036)	8(0.0011)	16(0.0087)
ko00920 Sulfur metabolism	30(0.0105)	15(0.0055)	16(0.0022)	6(0.0033)
ko00400 Phenylalanine, tyrosine and tryptophan biosynthesis	30(0.0105)	14(0.0051)	16(0.0022)	6(0.0033)
ko00030 Pentose phosphate pathway	28(0.0098)	13(0.0047)	5(0.0007)	12(0.0065)
ko00040 Pentose and glucuronate interconversions	27(0.0095)	7(0.0025)	12(0.0016)	6(0.0033)
ko00020 Citrate cycle	25(0.0088)	14(0.0051)	7(0.0010)	4(0.0022)
ko00300 Lysine biosynthesis	24(0.0084)	12(0.0044)	5(0.0007)	3(0.0016)
ko03020 RNA polymerase	16(0.0056)	7(0.0025)	25(0.0034)	2(0.0011)
ko00340 Histidine metabolism	15(0.0053)	6(0.0022)	10(0.0014)	7(0.0038)
ko00730 Thiamine metabolism	14(0.0049)	8(0.0029)	1(0.0001)	0(0.0000)
ko04610 Complement and coagulation cascades	14(0.0049)	5(0.0018)	43(0.0059)	4(0.0022)
ko00633 Nitrotoluene degradation	12(0.0042)	1(0.0004)	1(0.0001)	3(0.0016)
ko00312 beta-Lactam resistance	11(0.0039)	7(0.0025)	8(0.0011)	3(0.0016)
ko05150 Staphylococcus aureus infection	11(0.0039)	5(0.0018)	31(0.0042)	3(0.0016)

ko00600 Sphingolipid metabolism	10(0.0035)	6(0.0022)	17(0.0023)	6(0.0033)
ko00625 Chloroalkane and chloroalkene degradation	10(0.0035)	3(0.0011)	3(0.0004)	6(0.0033)
ko00660 C5-Branched dibasic acid metabolism	10(0.0035)	3(0.0011)	2(0.0003)	3(0.0016)
ko00983 Drug metabolism - other enzymes	9(0.0032)	3(0.0011)	6(0.0008)	2(0.0011)
ko00290 Valine, leucine and isoleucine biosynthesis	8(0.0028)	3(0.0011)	6(0.0008)	5(0.0027)
ko00460 Cyanoamino acid metabolism	8(0.0028)	2(0.0007)	2(0.0003)	4(0.0022)
ko00511 Other glycan degradation	7(0.0025)	2(0.0007)	5(0.0007)	3(0.0016)
ko00791 Atrazine degradation	6(0.0021)	1(0.0004)	12(0.0016)	3(0.0016)
ko00401 Novobiocin biosynthesis	6(0.0021)	4(0.0015)	2(0.0003)	1(0.0005)
ko04712 Circadian rhythm - plant	6(0.0021)	2(0.0007)	3(0.0004)	0(0.0000)
ko00592 alpha-Linolenic acid metabolism	5(0.0018)	2(0.0007)	10(0.0014)	3(0.0016)
ko00941 Flavonoid biosynthesis	4(0.0014)	2(0.0007)	8(0.0011)	1(0.0005)
ko00591 Linoleic acid metabolism	3(0.0011)	0(0.0000)	2(0.0003)	1(0.0005)
ko00785 Lipoic acid metabolism	3(0.0011)	2(0.0007)	9(0.0012)	2(0.0011)
ko00945 Stilbenoid, diarylheptanoid and gingerol biosynthesis	3(0.0011)	0(0.0000)	1(0.0001)	0(0.0000)
ko00965 Betalain biosynthesis	2(0.0007)	0(0.0000)	1(0.0001)	1(0.0005)
ko00942 Anthocyanin biosynthesis	1(0.0004)	0(0.0000)	0(0.0000)	0(0.0000)
ko03070 Bacterial secretion system	9(0.0032)	52(0.0189)	9(0.0012)	4(0.0022)
ko02040 Flagellar assembly	0(0.0000)	36(0.0131)	1(0.0001)	0(0.0000)
ko00564 Glycerophospholipid metabolism	13(0.0046)	27(0.0098)	45(0.0062)	11(0.0060)
ko03440 Homologous recombination	5(0.0018)	23(0.0084)	29(0.0040)	0(0.0000)
ko05111 Vibrio cholerae pathogenic cycle	3(0.0011)	21(0.0076)	9(0.0012)	4(0.0022)
ko00550 Peptidoglycan biosynthesis	5(0.0018)	15(0.0055)	12(0.0016)	6(0.0033)
ko00430 Taurine and hypotaurine metabolism	1(0.0004)	12(0.0044)	3(0.0004)	5(0.0027)
ko00480 Glutathione metabolism	6(0.0021)	11(0.0040)	16(0.0022)	5(0.0027)
ko00281 Geraniol degradation	2(0.0007)	11(0.0040)	0(0.0000)	3(0.0016)
ko00523 Polyketide sugar unit biosynthesis	1(0.0004)	6(0.0022)	0(0.0000)	0(0.0000)
ko00531 Glycosaminoglycan degradation	2(0.0007)	6(0.0022)	4(0.0005)	2(0.0011)
ko00471 D-Glutamine and D-glutamate metabolism	1(0.0004)	4(0.0015)	0(0.0000)	1(0.0005)
ko00642 Ethylbenzene degradation	2(0.0007)	4(0.0015)	5(0.0007)	2(0.0011)

ko00472 D-Arginine and D-ornithine metabolism	0(0.0000)	1(0.0004)	1(0.0001)	0(0.0000)
ko00524 Butirosin and neomycin biosynthesis	0(0.0000)	1(0.0004)	2(0.0003)	0(0.0000)
ko01100 Metabolic pathways	633(0.2219)	562(0.2047)	812(0.1110)	328(0.1777)
ko01120 Microbial metabolism in diverse environments	273(0.0957)	213(0.0776)	93(0.0127)	224(0.1213)
ko01110 Biosynthesis of secondary metabolites	214(0.0750)	200(0.0728)	216(0.0295)	128(0.0693)
ko02020 Two-component system	115(0.0403)	121(0.0441)	73(0.0100)	66(0.0358)
ko02010 ABC transporters	115(0.0403)	101(0.0368)	93(0.0127)	61(0.0330)
ko00230 Purine metabolism	68(0.0238)	62(0.0226)	96(0.0131)	19(0.0103)
ko00190 Oxidative phosphorylation	44(0.0154)	36(0.0131)	94(0.0128)	31(0.0168)
ko00520 Amino sugar and nucleotide sugar metabolism	39(0.0137)	52(0.0189)	26(0.0036)	10(0.0054)
ko00330 Arginine and proline metabolism	37(0.0130)	45(0.0164)	31(0.0042)	16(0.0087)
ko00640 Propanoate metabolism	27(0.0095)	21(0.0076)	11(0.0015)	14(0.0076)
ko00650 Butanoate metabolism	26(0.0091)	15(0.0055)	10(0.0014)	17(0.0092)
ko00260 Glycine, serine and threonine metabolism	25(0.0088)	37(0.0135)	11(0.0015)	11(0.0060)
ko00270 Cysteine and methionine metabolism	24(0.0084)	25(0.0091)	16(0.0022)	7(0.0038)
ko00630 Glyoxylate and dicarboxylate metabolism	22(0.0077)	22(0.0080)	8(0.0011)	18(0.0098)
ko00250 Alanine, aspartate and glutamate metabolism	19(0.0067)	17(0.0062)	18(0.0025)	6(0.0033)
ko00280 Valine, leucine and isoleucine degradation	18(0.0063)	19(0.0069)	12(0.0016)	12(0.0065)
ko00900 Terpenoid backbone biosynthesis	15(0.0053)	15(0.0055)	13(0.0018)	3(0.0016)
ko00710 Carbon fixation in photosynthetic organisms	13(0.0046)	10(0.0036)	10(0.0014)	2(0.0011)
ko00910 Nitrogen metabolism	13(0.0046)	23(0.0084)	11(0.0015)	6(0.0033)
ko00071 Fatty acid degradation	12(0.0042)	9(0.0033)	16(0.0022)	11(0.0060)
ko00053 Ascorbate and aldarate metabolism	12(0.0042)	9(0.0033)	10(0.0014)	6(0.0033)
ko00760 Nicotinate and nicotinamide metabolism	11(0.0039)	17(0.0062)	12(0.0016)	4(0.0022)
ko00770 Pantothenate and CoA biosynthesis	11(0.0039)	10(0.0036)	4(0.0005)	7(0.0038)
ko00790 Folate biosynthesis	11(0.0039)	12(0.0044)	11(0.0015)	1(0.0005)
ko00540 Lipopolysaccharide biosynthesis	10(0.0035)	23(0.0084)	1(0.0001)	0(0.0000)
ko00670 One carbon pool by folate	10(0.0035)	10(0.0036)	6(0.0008)	1(0.0005)
ko00130 Ubiquinone and other terpenoid-quinone biosynthesis	10(0.0035)	13(0.0047)	20(0.0027)	2(0.0011)
ko03430 Mismatch repair	10(0.0035)	14(0.0051)	19(0.0026)	1(0.0005)

ko00450 Selenocompound metabolism	10(0.0035)	9(0.0033)	5(0.0007)	4(0.0022)
ko00740 Riboflavin metabolism	9(0.0032)	11(0.0040)	10(0.0014)	1(0.0005)
ko04122 Sulfur relay system	8(0.0028)	7(0.0025)	5(0.0007)	1(0.0005)
ko02030 Bacterial chemotaxis	7(0.0025)	16(0.0058)	1(0.0001)	2(0.0011)
ko03060 Protein export	7(0.0025)	14(0.0051)	16(0.0022)	1(0.0005)
ko00830 Retinol metabolism	7(0.0025)	6(0.0022)	21(0.0029)	5(0.0027)
ko00440 Phosphonate and phosphinate metabolism	6(0.0021)	8(0.0029)	3(0.0004)	2(0.0011)
ko00950 Isoquinoline alkaloid biosynthesis	4(0.0014)	4(0.0015)	9(0.0012)	2(0.0011)
ko00521 Streptomycin biosynthesis	4(0.0014)	7(0.0025)	5(0.0007)	2(0.0011)
ko00960 Tropane, piperidine and pyridine alkaloid biosynthesis	4(0.0014)	4(0.0015)	8(0.0011)	3(0.0016)
ko00750 Vitamin B6 metabolism	4(0.0014)	8(0.0029)	2(0.0003)	0(0.0000)
ko00780 Biotin metabolism	4(0.0014)	10(0.0036)	5(0.0007)	1(0.0005)
ko00311 Penicillin and cephalosporin biosynthesis	2(0.0007)	3(0.0011)	2(0.0003)	2(0.0011)
ko00944 Flavone and flavonol biosynthesis	2(0.0007)	2(0.0007)	0(0.0000)	1(0.0005)
ko00901 Indole alkaloid biosynthesis	1(0.0004)	1(0.0004)	1(0.0001)	0(0.0000)

Table S12 Number of reads in QH and DQ related to two-component systems

KEGG module	Description	QH	DQ
M00434	PhoR-PhoB (phosphate starvation response) two-component regulatory system	321	471
M00458	ResE-ResD (aerobic and anaerobic respiration) two-component regulatory system	17	409
M00459	VicK-VicR (cell wall metabolism) two-component regulatory system	75	502
M00481	LiaS-LiaR (cell wall stress response) two-component regulatory system	10	241
M00490	MalK-MalR (malate transport) two-component regulatory system	4	10
M00492	LytS-LytR two-component regulatory system	50	31
M00499	HydH-HydG (metal tolerance) two-component regulatory system	49	32
M00500	AtoS-AtoC (cPHB biosynthesis) two-component regulatory system	44	93
M00505	KinB-AlgB (alginate production) two-component regulatory system	268	39
M00518	GlnK-GlnL (glutamine utilization) two-component regulatory system	8	214
M00519	YesM-YesN two-component regulatory system	29	130
M00447	CpxA-CpxR (envelope stress response) two-component regulatory system	73	33
M00454	KdpD-KdpE (potassium transport) two-component regulatory system	157	116
M00461	MtrB-MtrA (osmotic stress response) two-component regulatory system	13	26
M00501	PilS-PilR (type 4 fimbriae synthesis) two-component regulatory system	268	108

Table S13 Number of reads in QH and DQ related to ABC transporters

KEGG module	Description	QH	DQ
M00186	Tungstate transport system	127	42
M00191	Thiamine transport system	24	22
M00216	Multiple sugar transport system	22	517
M00198	Putative sn-glycerol-phosphate transport system	40	71
M00222	Phosphate transport system	1017	939
M00223	Phosphonate transport system	850	176
M00228	Putative glutamine transport system	100	391
M00239	Peptides/nickel transport system	992	2346
M00242	Zinc transport system	402	498
M00185	Sulfate transport system	583	139
M00436	Sulfonate transport system	1236	139
M00190	Iron(III) transport system	338	166
M00299	Spermidine/putrescine transport system	91	581
M00300	Putrescine transport system	913	141
M00209	Osmoprotectant transport system	258	36
M00237	Branched-chain amino acid transport system	1490	1859
M00240	Iron complex transport system	1291	1530
M00250	Lipopolysaccharide transport system	556	227
M00252	Lipooligosaccharide transport system	13	33

Table S14 Number of reads in QH and DQ related to osmotic stress response

Genes	QH	DQ
Glycine N-methyltransferase (EC 2.1.1.20)	0	2
Sarcosine N-methyltransferase	2	0
Dimethylglycine N-methyltransferase	2	1
Aspartokinase (EC 2.7.2.4) associated with ectoine biosynthesis	4	21
L-2,4-diaminobutyric acid acetyltransferase (EC 2.3.1.-)	13	2
Diaminobutyrate-pyruvate aminotransferase (EC 2.6.1.46)	41	26
L-ectoine synthase (EC 4.2.1.-)	12	11
Putative regulatory protein associated with the ectoine operon	4	2
Choline dehydrogenase (EC 1.1.99.1)	206	111
Betaine aldehyde dehydrogenase (EC 1.2.1.8)	108	52
High-affinity choline uptake protein BetT	316	84
Choline-sulfatase (EC 3.1.6.6)	128	50
HTH-type transcriptional regulator BetI	49	21
Glycine betaine transporter OpuD	58	168
Glycine betaine ABC transport system, ATP-binding protein OpuAA (EC 3.6.3.32)	66	2
Glycine betaine ABC transport system, permease protein OpuAB	84	2
Glycine betaine ABC transport system, glycine betaine-binding protein OpuAC	45	2
Glycine betaine ABC transport system, permease/glycine betaine-binding protein OpuABC	7	1
Glycine betaine/L-proline ABC transporter, glycine betaine/L-proline- binding/permease protein	50	1
Choline ABC transport system, ATP-binding protein OpuBA	10	1
Alcohol dehydrogenase GbsB (type III), essential for the utilization of choline (EC 1.1.1.1)	1	0
Osmotically activated L-carnitine/choline ABC transporter, substrate-binding protein OpuCC	4	0
Osmotically activated L-carnitine/choline ABC transporter, permease protein OpuCD	2	1

Osmotically activated L-carnitine/choline ABC transporter, permease protein OpuCB	11	3
Osmotically activated L-carnitine/choline ABC transporter, ATP-binding protein OpuCA	25	0
Sarcosine oxidase alpha subunit (EC 1.5.3.1)	239	39
Sarcosine oxidase beta subunit (EC 1.5.3.1)	153	18
Sarcosine oxidase delta subunit (EC 1.5.3.1)	0	1
Choline binding protein DCholine binding protein D	0	1
Choline binding protein A	3	1
GbcA Glycine betaine demethylase subunit AGbcA	108	3
